# Genomic Profiling Comparison of Germline *BRCA* and Non-*BRCA* Carriers Reveals *CCNE1* Amplification as a Risk Factor for Non-*BRCA* Carriers in Patients With Triple-Negative Breast Cancer

**DOI:** 10.3389/fonc.2020.583314

**Published:** 2020-10-30

**Authors:** Xin Huang, Di Shao, Huanwen Wu, Changbin Zhu, Dan Guo, Yidong Zhou, Chang Chen, Yan Lin, Tao Lu, Bin Zhao, Changjun Wang, Qiang Sun

**Affiliations:** ^1^Department of Breast Surgery, Peking Union Medical College Hospital, Beijing, China; ^2^BGI Genomics, BGI-Shenzhen, Shenzhen, China; ^3^Department of Pathology, Peking Union Medical College Hospital, Beijing, China; ^4^Clinical Biobank, Medical Science Research Center, Peking Union Medical College Hospital, Beijing, China

**Keywords:** triple negative breast cancer, *BRCA1*/2, *CCNE1*, genomic profiles, tumor mutation burden, microsatellite instability

## Abstract

**Background:** Differences in genomic profiling and immunity-associated parameters between germline *BRCA* and non-*BRCA* carriers in TNBC with high tumor burden remain unexplored. This study aimed to compare the differences and explore potential prognostic predictors and therapeutic targets.

**Methods:** The study cohort included 21 consecutive TNBC cases with germline *BRCA1/2* mutations and 54 non-*BRCA* carriers with a tumor size ≥ 2 cm and/or ≥1 affected lymph nodes. Differences in clinicopathological characteristics and genomic profiles were analyzed through next-generation sequencing. Univariate Kaplan–Meier analysis and Cox regression model were applied to survival analysis. Immunohistochemistry was used to confirm the consistency between *CCNE1* amplification and cyclin E1 protein overexpression.

**Results:** The cohort included 16 and five patients with germline *BRCA1* and *BRCA2* mutations, respectively. Patients with germline *BRCA1/2* mutations were diagnosed at a significantly younger age and were more likely to have a family history of breast and/or ovarian cancer. Six non-*BRCA* carriers (11.11%) carried germline mutations in other cancer susceptibility genes, including five mutations in five homologous recombination repair (HRR) pathway genes (9.26%) and one mutation in *MSH3* (1.85%). Somatic mutations in HRR pathway genes were found in 22.22 and 14.29% of the non-*BRCA* and *BRCA* carriers, respectively. *PIK3CA* missense mutation (*p* = 0.046) and *CCNE1* amplification (*p* = 0.2) were found only in the non-*BRCA* carriers. The median tumor mutation burden (TMB) was 4.1 Muts/Mb, whereas none of the cases had high microsatellite instability (MSI). *BRCA* status did not affect disease-free survival (DFS, *p* = 0.15) or overall survival (OS, *p* = 0.52). *CCNE1* amplification was an independent risk factor for DFS in non-*BRCA* carriers with TNBC (HR 13.07, 95% CI 2.47–69.24, *p* = 0.003). Consistency between *CCNE1* amplification and cyclin E1 protein overexpression was confirmed with an AUC of 0.967 for cyclin E1 signal intensity.

**Conclusions:** We found differences in genetic alterations between germline *BRCA* and non-*BRCA* carriers with TNBC and a high tumor burden. TMB and MSI may not be suitable predictors of TNBC for immune checkpoint inhibitors. Notably, *CCNE1* amplification is a novel potential prognostic marker and therapeutic target for non-*BRCA* carriers with TNBC. Cyclin E1 may be used instead of *CCNE1* to improve clinical applicability.

## Introduction

Triple-negative breast cancer (TNBC) has been defined as a subtype of breast cancer negatively expressing estrogen receptor (ER), progesterone receptor (PR), and human epidermal growth factor receptor 2 (HER2) (1), accounting for ~15–20% of newly diagnosed breast cancer (2). Women with TNBC have a higher rate of early distant recurrence and worse 5-years prognosis than those with other subtypes of breast cancer (3, 4).

Hereditary breast cancer differs from sporadic breast cancer because of the impact of germline genetic variation. Testing for germline mutations in breast cancer predisposition genes has become a standard practice (5). *BRCA*-associated breast cancer is the most common type of hereditary breast cancer (6, 7). In addition to *BRCA1/BRCA2*, some other germline mutations in high- and moderate-penetrance genes play significant roles in increasing breast cancer risk for mutation carriers (8). However, testing for other breast cancer predisposition genes such as *TP53, PTEN*, and *PLAB2* is still required to determine family history or specific clinical features (9, 10) compared with other cancer predisposition genes. Not all patients with germline mutations have a known family cancer history or specific clinical features, which results in about 50–80% of at-risk individuals not being successfully identified as such (11). Thus, these criteria alone may not provide sufficient information for testing and assessment of other genetic cancer predisposition genes (12) in non-*BRCA* carriers with TNBC.

Recently, next-generation sequencing (NGS) has been increasingly used in cancer risk assessment in clinical practice (13). Sequencing with multigene panels may identify significant differences to further understand the relationship between genetic profiling and tumor biology (14). TNBC is highly intertumorally and intratumorally heterogeneous and incorporates various molecular, and clinical pathological features and distinct clinical outcomes (15). Hence, sequencing with multigene panels can be used to recognize the heterogeneity of TNBC through genomic profiling.

The POSH study (16) showed the early survival advantage of patients with *BRCA* mutation carriers with TNBC, who have a lower likelihood of dying from early breast cancer than non-*BRCA* carriers. This advantage might reflect the higher sensitivity of DNA repair deficiency associated with *BRCA*-mutant breast cancers to chemotherapy, particularly that of a higher response to platinum-based drugs (17, 18) or the greater visibility of *BRCA*-mutant breast cancers to host immune attack (19). Although poly-ADP-ribose polymerase (PARP) inhibitors displayed clinical efficiency for approved *BRCA*-mutant patients with TNBC (20), the potential targeted therapeutic treatments for non-*BRCA* carriers with TNBC need to be explored considering the growing evidence associating germline mutations with cancer predisposition as well as the availability of targeted therapies (21–23).

Tumor mutation burden (TMB) (24) and microsatellite instability (MSI) (25) have been extensively investigated in breast cancer. Given the lack of effective targeted therapies for TNBC, immunotherapeutic approaches and associated predictive markers, such as TMB, MSI, and programmed cell death ligand 1 (PD-L1) (26), remain a focus of great interest. Romualdo et al. (27) found that a high TMB was associated with clinical benefit in patients with metastatic TNBC receiving anti-programmed death 1(PD-1)/PD-L1 therapy. Yoshiya et al. (28) evaluated MSI in 63 patients with TNBC exhibiting a high number of tumor-infiltrating lymphocytes and found that MSI-high (MSI-H) tumors were absent among those with enriched PD-L1 responding to immunotherapy. There are currently no specific guidelines for assessing TMB and MSI in TNBC. In addition, a high TMB and MSI-H tumors are not uncommon in patients with TNBC, who are potential candidates for treatment with immune checkpoint inhibitors (ICIs) (29). It remains necessary to study the role of TMB and MSI (30)in relation to immunotherapy in Chinese patients with TNBC.

Previous studies have reported the overall genomic landscape of unselected (without any specific tumor characteristics) TNBC cases in Chinese (14, 31, 32), American, and European populations (32–34). For example, Jiang (31) classified TNBCs into four transcriptome-based subtypes by comprehensively analyzing the clinical, genomic, and transcriptomic data of a cohort of 465 unselected primary triple-negative breast cancer patients. However, the genomic profiles of selected TNBC cases with specific tumor characteristics such as large tumor size or involved lymph nodes remain to be explored. In addition, these studies lack data about differences in genomic profiles via the comparison between germline *BRCA* and non-*BRCA* carriers. Even Chen et al. (35) mainly identified the differences in somatic mutation profiles between *BRCA*, non-*BRCA* germline mutation, and non-carriers with unselected breast cancer not focused on TNBC. Immunity-associated parameters, potential predictors, and therapeutic targets for non-*BRCA* carriers with TNBC were not included in these studies, either. Furthermore, TNBCs with a high tumor burden were associated with worse prognosis (36) and required the identification of distinct prognostic predictors and potential therapeutic targets.

In the present study, we performed a capture-based, targeted NGS utilizing a panel comprising 508 cancer-associated genes in Chinese patients with TNBC with high tumor burden. We aimed to identify potential prognostic and therapeutic markers in non-*BRCA* carriers with TNBCs exhibiting a high tumor burden by comparing differences in genomic profiling together with immunity-associated parameters in patients with different germline *BRCA* status.

## Methods

### Ethics

All the procedures performed in this study involving human participants were conducted following the ethical standards of the institutional and national research committees and with the 1964 Declaration of Helsinki and its later amendments or comparable ethical standards. The study was approved by the Ethics Committee of Peking Union Medical College Hospital (No. HS-1623), and written informed consent was obtained from all participants.

### Patients and Specimens

Triple negative breast cancer patients that met the eligibility criteria including T ≥ 2 cm and/or number of affected lymph nodes ≥1 were acquired from Peking Union Medical College Hospital between October 2013 and April 2019 and were selected to form a consecutive cohort including 87 primary TNBC patients. Triple negative breast cancer was defined as negative ER, negative PR and negative human epidermal growth factor receptor-2 (HER2). ER, PR, HER2, and other receptors in each specimen were routinely evaluated by immunohistochemistry (IHC) staining at the Department of Pathology in Peking Union Medical College Hospital. HER2 status was confirmed by IHC and/or fluorescence *in situ* hybridization according to the 2018 American Society of Clinical Oncology/College of American Pathologists Clinical Practice guidelines (37). We excluded patients with a diagnosis of other malignant tumors such as gastric, lung, and ovarian cancer.

Primary tumor formalin-fixed and paraffin-embedded (FFPE) tissue and paired peripheral blood samples were obtained along with their clinical and therapeutic information in the same batch. NGS with a panel of 508 cancer-associated genes was applied to both specimens and blood samples within 1 year after the initial treatment. Clinicopathological characteristics and treatment regimen, including age; T stage; histological grade; TNM stage; lymph node status; lymphovascular invasion (LVI); ER/PR/HER2 status; and treatment variables, including surgery, chemotherapy, and radiation therapy, were collected.

### Study Design and Endpoints

Patients with TNBC were allocated to groups according to the germline *BRCA1/2* mutation status. Disease-free survival (DFS) was defined as the time from the date of surgery until first disease recurrence at a local, regional, or distant site or the diagnosis of contralateral breast cancer. Overall survival (OS) was defined as the time from the date of surgery until death from any cause. Patients without any events were censored at the date of last follow-up.

### Targeted Exome Sequencing of TNBCs

Genomic DNA (gDNA) was extracted from FFPE and peripheral blood samples using the Qiagen DNeasy Blood & Tissue Kit (Qiagen, Hilden, Germany) as per the manufacturer's protocol. DNA concentration and quality were assessed by Qubit (Life Technologies, Gaithersburg, MD, USA) and agarose gel electrophoresis. gDNA (250 ng) was used for sequence library construction using a previously described method (38). The hybridization product was subsequently purified, amplified, and quantified. Finally, sequencing of 508 key cancer-related genes was performed with a paired-end 100 and 8 bp barcodes on a MGISEQ-2000 sequencer following the manufacturer's protocol.

Raw data were first filtered by SOAPnuke to exclude low-quality reads. Clean reads were then aligned to the reference human genome (UCSC hg19) using the BWA MEM algorithm. Single-nucleotide variants were detected by the Genome Analysis Toolkit (GATK) Unified Genotyper. Small insertions and deletions were identified using GATK Haplotype. Copy number variations (CNVs) were identified using read-depth analysis. All above variants were further filtered by quality depth, strand bias, mapping quality, and read position. Each variant was finally annotated with respect to gene location.

All deleterious germline mutations were confirmed by Sanger sequencing in duplicate samples. Pathogenic mutations were defined as those leading to a truncated protein or those that have been previously reported to be associated with disease.

### Analysis of MSI

MSI analysis was determined by next generation sequencing of 16 microsatellite loci, including BAT25, BAT26, NR24, D2S123, D5S346, NR21, MONO27, BAT40, BAT34c4, D18S55, D1S2883, D17S261, D17S799, D18S35, D18S58, and D17S250 (39–41). The sequences were compared with matched peripheral blood samples. The mSINGS, MSISensor, and MANTIS algorithms were used to determine if extent of the detected instability was significant, and the samples were categorized as MSI-H, MSI-low, or microsatellite stable.

### Immunohistochemistry (IHC)

Cyclin E antibody (clone HE12) was obtained from Milipore (Temecula, CA, USA). FFPE tissue sections were deparaffinized and hydrated. Antigen retrieval was performed using pH9 Antigen Retrieval Solution (DAKO). Peroxide blocking was performed using 3% H_2_O_2_ in methanol at room temperature for 5 min. The slides were incubated with primary antibody for 30 min for all antibodies at a concentration of Cyclin E 1:2,000. EnVision Flex+ (DAKO) was used as the detection system following the manufacturer's instructions and was developed using freshly prepared 0.05% 3′,3-diaminobenzidine tetrahydrochloride.

Finally, the slides were counterstained with hematoxylin, dehydrated, and mounted. Positive and negative controls of placenta tissues were performed in each run. Cyclin E immunohistochemical expression was quantified by two independent pathologists who were blinded to the identity of the samples using a four-value intensity score (0, 1+, 2+, and 3+), expression score (H score), and the percentage extent of reactivity. A consensus value on both intensity and extension was reached by the two independent observers. A final consensual score was obtained by multiplying both intensity and extension values (range, 0–300) (42).

### Comparison of *CCNE1* Status Using Public Databases

Difference of *CCNE1* amplification between patients with/without TNBC was compared via the Molecular Taxonomy of Breast Cancer International Consortium (METABRIC) (43) and the Cancer Genome Atlas (TCGA) (44) datasets. All *CCNE1* profiling data from METABRIC or TCGA was obtained from analyzed NGS data provided by cBioPortal (www.cbioportal.org) (45). *CCNE1* expression of 150 samples from METABRIC, including 131 TNBC cases with normal *CCNE1* expression and 19 TNBC cases with *CCNE1* amplification, were used for comparison of OS.

### Statistical Analysis

Categorical variables were analyzed by Pearson's chi-square test or Fisher's exact test. Kaplan–Meier (K–M) curves were used to display DFS and OS. Log-rank test was applied to test survival difference among groups with various genomic and clinical characters. Cox uni- and multivariate analyses were used to construct the risk model that predicted disease recurrence as well as cancer-related death. To determine whether IHC staining for cyclin E1 could be used instead of genetic testing for *CCNE1*, the consistence was assessed by calculating area under the ROC curve (AUC) with bootstrap correction.

## Results

### Study Population

A total of 87 Chinese female patients with TNBC who met the eligibility criteria including a tumor size of ≥2 cm and/or ≥1 affected lymph nodes were included in the study. By NGS, the prevalence of germline *BRCA1/2* mutation was 24.1% (21/87) in the study cohort. Five patients with a diagnosis of other malignant tumors including gastric carcinoma (*n* = 2), lung cancer (*n* = 2), and ovarian cancer (*n* = 1) were excluded from the study. Furthermore, three patients with incomplete medical records and four patients who were lost to follow-up were excluded. Finally, 21 germline *BRCA* carriers and 54 non-*BRCA* carriers were enrolled in the study (Supplementary Figure 1). The median follow-up was 30 months (range, 6–66 months), with a 4.6% of the patients lost to follow-up. The DFS and OS rates in *BRCA1/2* and non-*BRCA* carriers are shown in Supplementary Table 2.

### Patient Characteristics and Treatment History

We first analyzed the clinicopathological characteristics to understand predisposing factors associated with germline *BRCA1/2* mutations in TNBC. Breast cancer was diagnosed at a significantly younger age in germline *BRCA* carriers than in non-*BRCA* carriers (*p* = 0.007, Table 1). Moreover, germline *BRCA1/2* carriers were more likely to have a family history of breast and/or ovarian cancer (*p* = 0.008, Table 1). However, other clinical characteristics showed no further significant correlations (Supplementary Table 1) between germline *BRCA1/2* mutation status and other clinicopathological factors, including histology (*p* = 0.83), T stage (*p* = 0.65), lymph node status (*p* = 0.93), histological grade (*p* = 0.09), LVI (*p* = 0.44), and Ki-67 index (*p* = 0.48), the same to surgical management of breast and axilla (*p* = 0.88 and 0.53, respectively) and systemic treatment including chemotherapy and radiotherapy (*p* = 0.48 and 0.46, respectively).

**Table 1:** Clinicopathological characteristics of Chinese female patients with triple-negative breast cancer according to *BRCA* germline mutation status in this study cohort (*p* < 0.05).

		**No. (%)**		
**Characteristics (*N* = 75)**	**Parameter**	***BRCA* germline mutation**	***BRCA* non-carrier**	***p*-value**
Age, years	≤ 35	8 (38.1)	14 (25.9)	
	36–45	11 (52.4)	12 (22.2)	
	46–55	1 (4.8)	18 (33.4)	
	>55	1 (4.7)	10 (18.5)	
				0.007
Family history	None	11 (52.4)	45 (83.3)	
	*BRCA*-related	9 (42.9)	6 (11.1)	
	Non-*BRCA*-related	1 (4.7)	3 (5.6)	
				0.008

*P-values were derived from the Pearson's Chi-square test, Fisher's exact test and Continuity Correction chi-square test*.

### Overall Landscape of Germline *BRCA* Mutation and Somatic Mutation in This Chinese Cohort

Twenty-one patients were found carrying *BRCA1/2* mutations in individuals (Supplementary Figure 2B). Details for germline *BRCA1/2* mutations were shown in Table 2, in which no synonymous variation was found in all single nucleotide variants (SNV).

**Table 2:** *BRCA* mutation in details of 21 patients with TNBC in the cohort.

**Patient no**.	**Gene_symbol**	**c.HGVS**	**p.HGVS**	**Variation type**	**Variation type**	**Synounymous or Non-synounymous**
3	*BRCA2*	c.7975A>G	p.R2659G	SNV	Missense	Non-synounymous
8	*BRCA1*	c.2572C>T	p.Q858^*^	SNV	Nonsense	Non-synounymous
12	*BRCA1*	c.4698_4704del TGGAATC	p.G1567Afs^*^32	Indel	Frameshift	/
32	*BRCA2*	c.3860delA	p.N1287Ifs^*^6	Indel	Frameshift	/
45	*BRCA1*	c.4801A>T	p.K1601^*^	SNV	nonsense	Non-synounymous
46	*BRCA1*	c.5521delA	p.S1841Vfs^*^2	Indel	Frameshift	/
47	*BRCA2*	c.3085_3087delATGinsTA	p.M1029Yfs^*^14	Indel	Frameshift	/
48	*BRCA1*	c.17_18delTT	p.L6Pfs^*^3	Indel	Frameshift	/
49	*BRCA1*	c.441+2T>A	/	splice	Splice	/
50	*BRCA1*	c.2751delC	p.K918Sfs^*^82	Indel	Frameshift	/
55	*BRCA1*	c.5470_5477del	p.l1824Dfs^*^3	Indel	Frameshift	/
58	*BRCA1*	c.4222C>T	p.Q1408^*^	SNV	Nonsense	Non-synounymous
62	*BRCA1*	c.4756G>T	p.E1586^*^	SNV	nonsense	Non-synounymous
63	*BRCA1*	c.3756_3759delGTCT	p.S1253Rfs^*^10	Indel	Frameshift	/
65	*BRCA2*	c.2059_2063del	p.D687^*^	Indel	Frameshift	/
67	*BRCA1*	c.5470_5477delATTGGGCA	p.I1824Dfs^*^3	Indel	Frameshift	/
69	*BRCA1*	c.5470_5477delATTGGGCA	p.I1824Dfs^*^3	Indel	Frameshift	/
70	*BRCA2*	c.9122C>G	p.Ser3041^*^	SNV	Nonsense	Non-synounymous
71	*BRCA1*	c.3G>T	p.0	SNV	Start loss	/
72	*BRCA1*	c.2572C>T	p.Q858^*^	SNV	Nonsense	Non-synounymous
73	*BRCA1*	c.4801A>T	p.K1601^*^	SNV	Nonsense	Non-synounymous

*SNV, single nucleotide variant; Indel, insertion-deletion*.

The average target coverage depths were ≥500 × for all specimens and blood samples. Among the TNBCs with genomic profiling data, 480 somatic mutations were identified (Supplementary Figure 2A), comprising 13 Delins (2.71%), 48 Frameshifts (10.00%), 253 Missenses (52.71%), 38 Nonsenses (7.92%), 17 Splices (3.54%), one Start-Alt (0.21%), and 110 CNVs (22.9%), including 102 copy number gains (21.25%) and eight copy number losses (1.67%). Copy number variations (CNVs) were identified by comparing sequence coverage of targeted regions in a tumor sample relative to the normal sample using CONTRA (46). First, read-depth statistics (log-ratio) were calculated from baits originating in the same exon. Then, Adjacent exons were merged into larger segments if the read depths of their component baits were not significantly different by *t*-test, and log-ratio were recalculated for the larger segments. We call segment a CNV event, if the segment has a log-ratio >0.3 (gain) or < -0.3 (loss). CNV analysis identified an average of 0.1 (range, 0–3) CNV loss and 1.4 (range, 0–10) amplified genes per patient (Supplementary Figure 2B).

### Comparison of the Genomic Profiles of Germline *BRCA* and Non-*BRCA* Carriers With TNBC in a Chinese Cohort

#### Comparison of Germline Mutations

In the *BRCA* carrier group, 16 and five patients harbored *BRCA1* and *BRCA2* mutations, respectively. Conversely, in the non-*BRCA* carrier group, six patients (11.11%, 6/54) harbored a total of six mutations in other cancer susceptibility genes beyond *BRCA1/2*. Of these, five mutations (9.26%, 5/54) were in homologous recombination repair (HRR) pathway genes, including *PALB2* (1.85%, 1/54), *BLM* (1.85%, 1/54), *NBN* (1.85%, 1/54), *RAD51C* (1.85%, 1/54), and *RAD51D* (1.85%, 1/54), and one mutation was in *MSH3* (1.85%, 1/54) related to Lynch syndrome. None of the patients harbored simultaneous *BRCA* mutations and other germline mutations (*p* = 0.17). Interestingly, among these eight genes, three belonged to the Fanconi anemia gene family, including *BRCA2, PALB2*, and *RAD51C*, with 25.92% (7/27) of patients carrying germline mutations in any of these genes.

#### Comparison of Somatic Mutations

**Figure 1:**
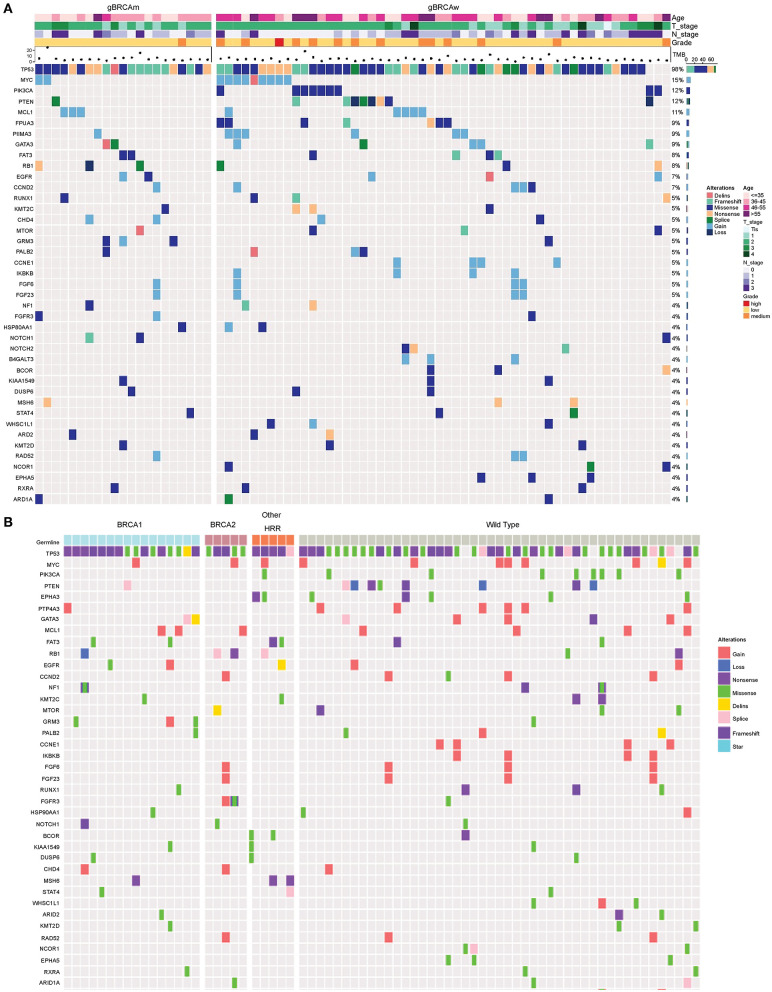
Somatic mutation spectra among different groups. **(A)** Somatic mutation spectrum between germline *BRCA* and non-*BRCA* carriers (mutation frequency equal to or more than 4% in the whole cohort). **(B)** Somatic mutation spectrum among *gBRCA1, gBRCA2*, other gHRR, and non-gHRR carriers. Each column represents a patient and each row represents a gene. In **(A)**, the number on the right represents the percentage of patients with mutations in a specific gene in the whole cohort. The top plot represents the overall number of mutations detected in a patient. Different colors denote different types of mutation. The annotation at the top depicts the germline mutations carried by the patients. HRR, homologous recombination repair.

Genomic alterations with variation allele frequency ≥4% were listed (Figure 1A) to understand the intertumoral heterogeneity between the two TNBC subgroups. No somatic *PIK3CA* missense was detected in any of the patients with germline *BRCA1/BRCA2* mutations (Table 3, *p* = 0.046). *GRM3* mutation was found more in *BRCA* carriers (Table 3, *p* = 0.03). In addition, several somatic mutations also showed non-significantly enriching tendencies in non-*BRCA* carriers, including *NOTCH2, B4GALT3, BCOR, WHSC1L1, NCOR1*, and *EPHA5* (Supplementary Table 3). Interestingly, somatic *CCNE1* and *IKBKB* amplification in patients with TNBC were mutually exclusive with germline *BRCA1/BRCA2* mutation (*p* = 0.20 and 0.20, respectively, Supplementary Table 3) as well as other germline-mutated genes involved in the homologous recombination repair (HRR) pathway (*PALB2, BLM, NBN, RAD 51C*, and *RAD 51D*) (Figure 1B), although there was no statistical significance.

**Table 3:** Comparison of somatic mutations between *BRCA* germline mutation carriers and non-carriers of triple-negative breast cancer (mutation frequency equal to or more than 4% in the whole cohort, *p*-value < 0.05).

	**mut (*N* = 21)**	**wild (*N* = 54)**	**Total (*N* = 75)**	***p*-value**
*PIK3CA*				0.046
mut	0 (0.0%)	9 (16.7%)	9 (12.0%)	
Wild	21 (100.0%)	45 (83.3%)	66 (88.0%)	
*EPHA3*				0.08
mut	0 (0.0%)	7 (13.0%)	7 (9.3%)	
Wild	21 (100.0%)	47 (87.0%)	68 (90.7%)	
*GRM3*				0.03
mut	3 (14.3%)	1 (1.9%)	4 (5.3%)	
Wild	18 (85.7%)	53 (98.1%)	71 (94.7%)	

*mut, mutation*.

*P-values were derived from the Pearson's Chi-square test, Fisher's exact test and Continuity Correction chi-square test*.

**Figure 2:**
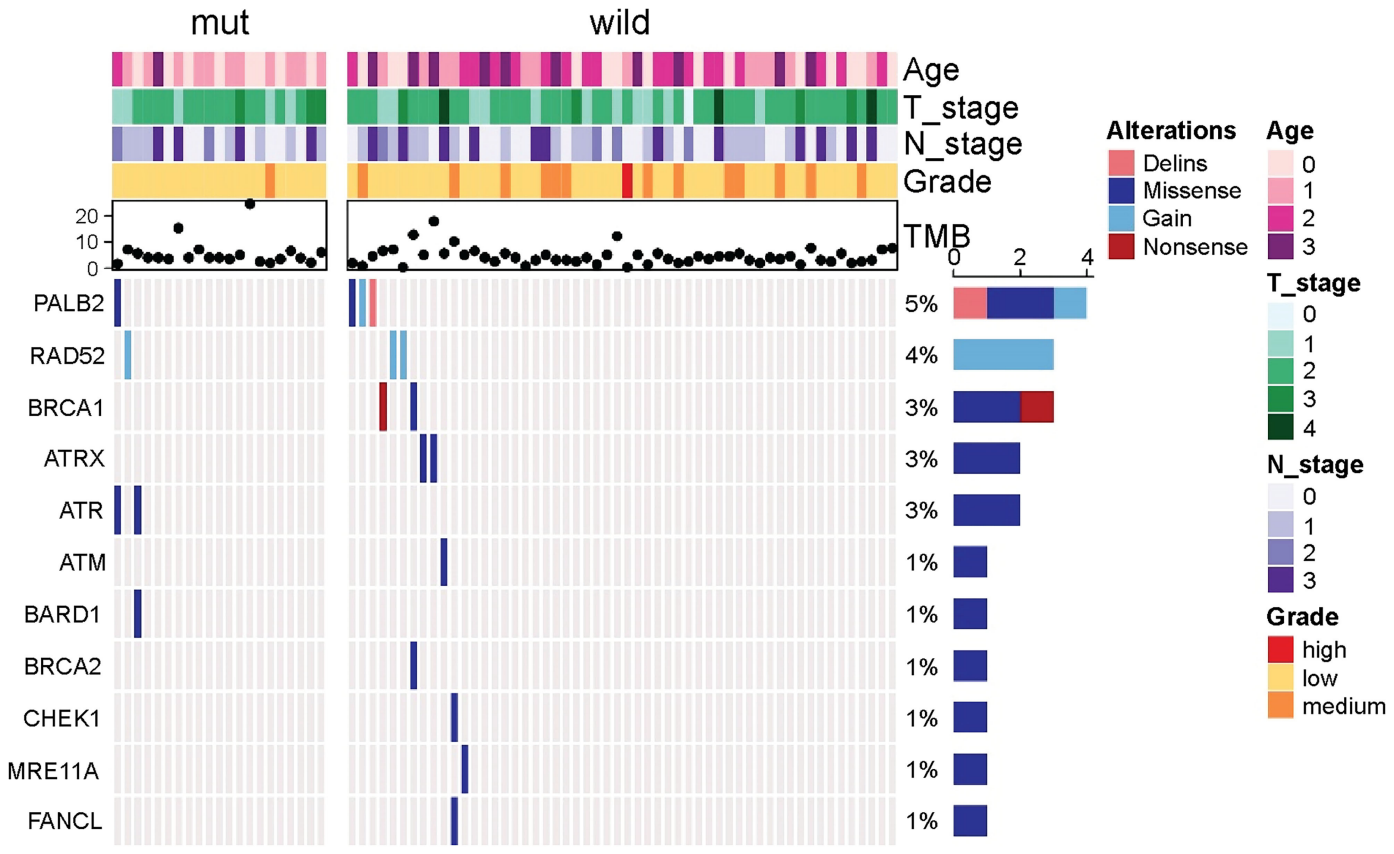
Comparison of somatic mutant genes involved in the homologous recombination repair pathway including somatic *BRCA* mutations between germiline *BRCA* and non-*BRCA* carriers.

Because other germline mutations in HRR pathway genes were very common in non-*BRCA* carriers, somatic mutations were also compared due to different germline *BRCA* status. In this study cohort, 19 somatic mutations in 12 genes were involved in the HRR pathway from 15 patients (20.0%, 15/75) including *ATRX, ATM, ATR, BARD1, BRCA1, BRCA2, CHEK1, MRE11A, PALB2, RAD52*, and *FANCL*. In particular, *PALB2* dominated in four patients (26.67%, 4/15) (Figure 2). There were three *BRCA* carriers (3/21, 14.29%) accompanying somatic HRR-mutant genes compared to 12 non-*BRCA* carriers (22.22%, 12/54) (*p* = 0.53, Supplementary Table 4). *ATR* (*p* = 0.02) was only detected in *BRCA* carriers (Table 4). In contrast, *ATRX, ATM, CHEK1, MRE11A*, and *FANCL* were only found in non-*BRCA* carriers even without statistical significance (all *p* = 0.53, Supplementary Table 4). One patient with germline *BRCA1* mutation had co-occurring somatic *ATR* and *PALB2* mutations, while another patient with germline *BRCA2* mutation had co-occurring somatic *ATR* and *BARD1* mutations and a third patient with germline *BRCA2* mutation had co-occurring somatic *RAD52* mutation. No concomitant germline and somatic *BRCA* or other HRR gene mutations occurred in any other patients. Moreover, missense mutation dominated in somatic mutation types involved in HRR pathway genes (81.48%, 22/27, Figure 2).

**Table 4:** Comparison of somatic mutant genes involved in the homologous recombination repair pathway between *BRCA* germline mutation carriers and non-carriers of triple-negative breast cancer (*p* < 0.05).

	**mut (*N* = 21)**	**wild (*N* = 54)**	**Total (*N* = 75)**	***p-*value**
*ATR*				0.02
mut	2 (9.5%)	0 (0.0%)	2 (2.7%)	
Wild	19 (90.5%)	54 (100.0%)	73 (97.3%)	

*mut, mutation*.

*P values were derived from the Pearson's Chi-square test, Fisher's exact test and Continuity Correction chi-square test*.

### Comparison of TMB and MSI Between Germline *BRCA* and Non-*BRCA* Carriers in Chinese Patients With TNBC

Median TMB (4.1 Muts/Mb) remained the same in both germline *BRCA* and non-*BRCA* carriers with TNBC in this study cohort (*p* = 0.38), with a range of 1.79–24.62 Muts/Mb for the former and 0.51–17.95 Muts/Mb for the latter. Interestingly, TMB of the 4 patients with *CCNE1* amplification were all lower than 4.1 Muts/Mb.

Only one case of low MSI was detected in germline *BRCA* carriers compared with two cases in non-*BRCA* carriers (*p* = 0.63). The rest belonged to microsatellite stability, and no MSI-H cases were found in this study cohort.

### Survival Analysis

#### Survival Analysis Between Germline *BRCA* and Non-*BRCA* Carriers With TNBC

**Figure 3:**
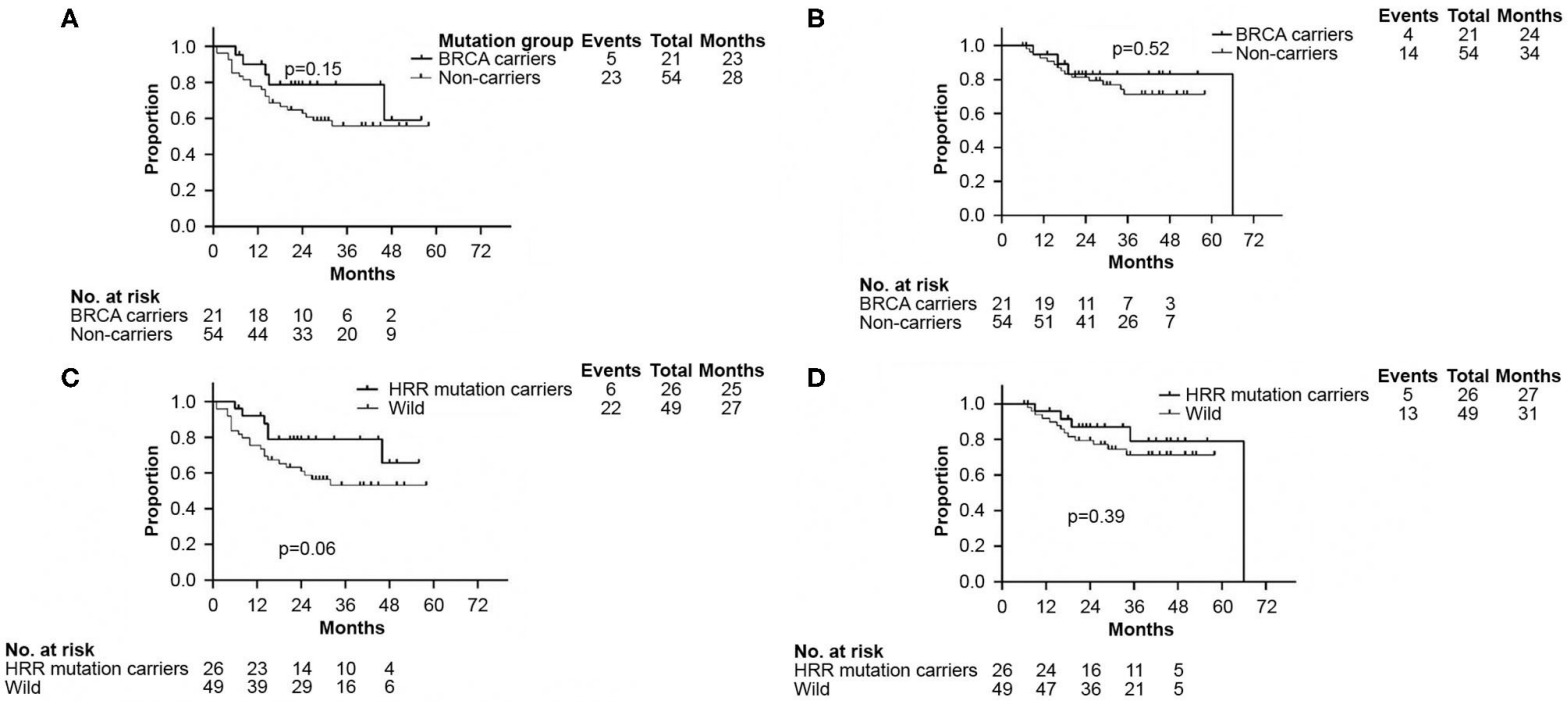
Kaplan–Meier analyses performed to confirm neither *BRCA* germline mutation status nor HRR germline mutation status affected DFS and OS. **(A)** Kaplan–Meier curve of DFS according to *BRCA* germline mutation status. **(B)** Kaplan–Meier curve of OS according to *BRCA* germline mutation status. **(C)** Kaplan–Meier curve of DFS according to genes involved in HRR pathway mutation status. **(D)** Kaplan–Meier curve of OS according to genes involved in HRR pathway mutation status. DFS, disease free survival; OS, overall survival. HRR, homologous recombination repair.

There were no significant differences in DFS and OS between the germline *BRCA* and non-*BRCA* carriers with TNBC (*p* = 0.15 and *p* = 0.52, respectively) (Figures 3A,B). In addition, germline mutations involving HRR pathway genes did not affect either DFS or OS too (*p* = 0.06 and *p* = 0.39, respectively, Figures 3C,D).

#### Survival Analysis and Risk Factors for DFS and OS in Non-*BRCA* Carriers With TNBC

**Figure 4:**
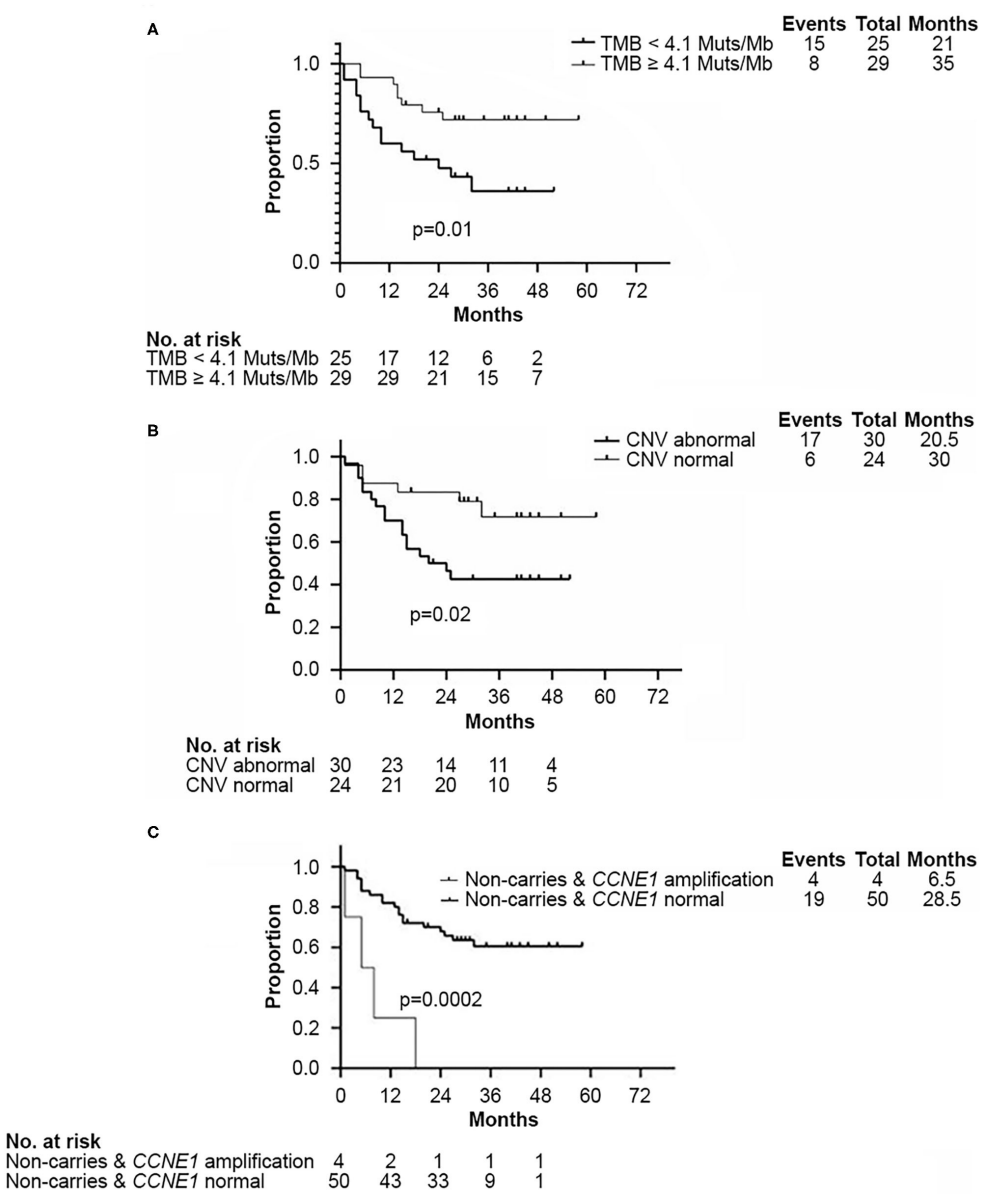
Kaplan–Meier analysis showed disease-free survival in non-*BRCA* carriers with TNBC in this cohort. **(A)** Kaplan–Meier curve of DFS according to median TMB (4.1 Muts/Mb) in this cohort. **(B)** Kaplan–Meier curve of DFS according to CNV status. **(C)** Kaplan–Meier curve of DFS according to *CCNE1* CNV status. DFS, disease free survival; CNV, copy number variation; TMB, tumor mutation burden.

Survival analysis showed that both TMB <4.1 Muts/Mb and abnormal CNV were associated with worse DFS (*p* = 0.01 and *p* = 0.02, respectively, Figures 4A,B). Approximately 80% (102/120) of abnormal CNVs belonged to CNV gain (Supplementary Figure 2A). As mentioned above, the TMB value of those four patients with *CCNE1* amplification were all found lower than 4.1 Muts/Mb. K–M curves showed that DFS in patients with TNBC was affected by *CCNE1* amplification (*p* = 0.0002, Figure 4C). Univariate analysis for correlation of DFS and OS was applied to clinicopathological characteristics, TMB, CNV, HRR pathway genes, high-frequency somatic mutation genes, and genes with somatic mutation detected only in non-*BRCA* carriers (Supplementary Tables 5, 6). The results revealed that advanced T stage (*p* = 0.007) and TNM stage (*p* = 0.02), low TMB (*p* = 0.01), abnormal CNV (*p* = 0.03), *GATA3* mutation (*p* = 0.009), and *CCNE1* amplification (*p* = 0.001) were associated with worse DFS (Supplementary Table 5), whereas advanced T stage (*p* < 0.001), N stage (*p* = 0.01), and TNM stage (*p* = 0.01) were associated with worse OS (Supplementary Table 6).

**Figure 5:**
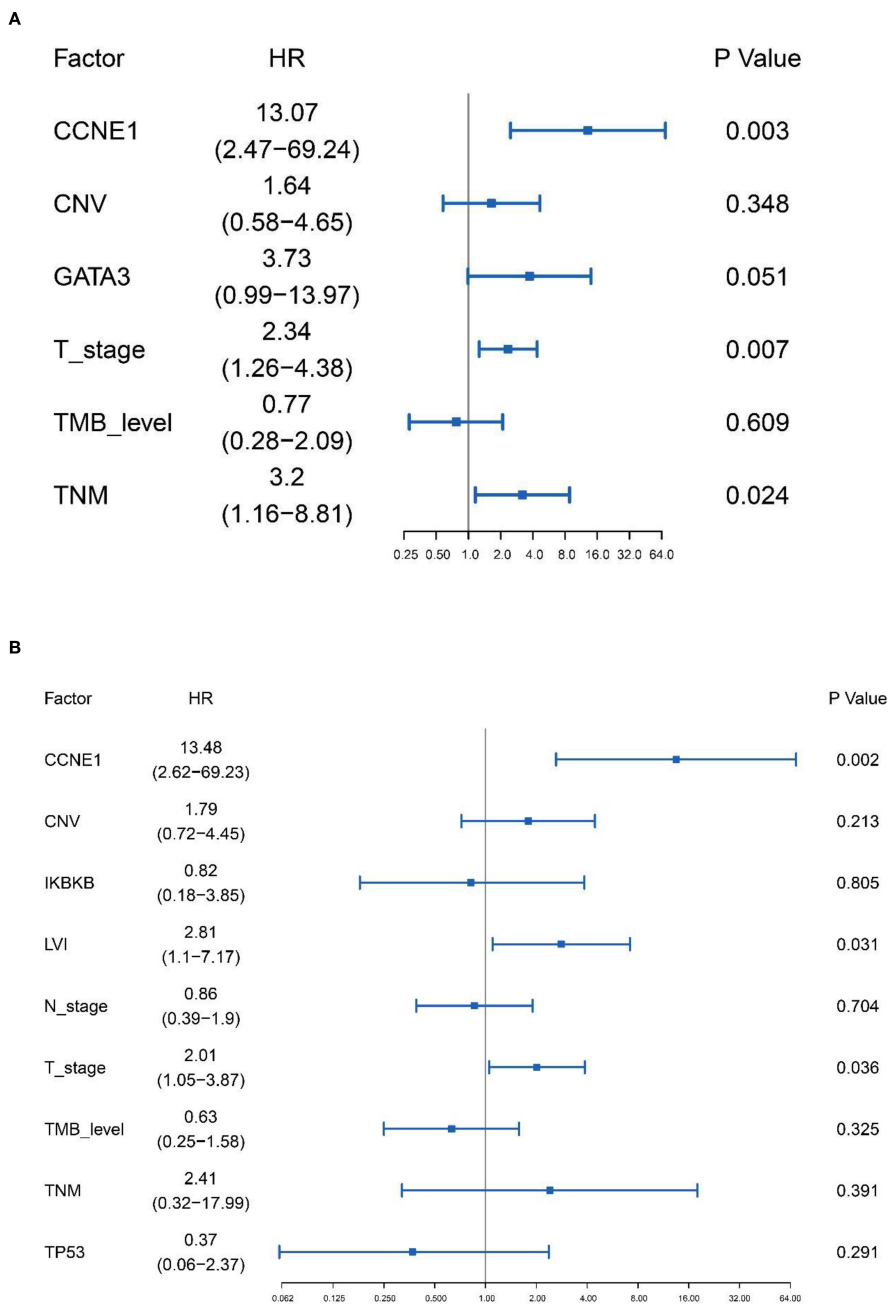
Cox proportional hazards regression model performed to determine the risk factors associated with disease-free survival in non-*BRCA* carriers with TNBC and all TNBC patients in this cohort. **(A)** Cox proportional hazards regression model for non-*BRCA* carriers with TNBC. **(B)** Cox proportional hazards regression model for all TNNC patients. HR, hazard ratio; CNV, copy number variation; TMB, tumor mutation burden.

Based on the results of univariate analysis, Cox regression modeling was performed to evaluate the risk factors associated with DFS and OS in non-*BRCA* carriers. We found that T stage, TNM stage, and *CCNE1* amplification were the independent risk factors for DFS [T stage, HR = 2.34 (95% CI, 1.26–4.38), *p* = 0.007; TNM stage, HR = 3.20 (95% CI, 1.16–8.81), *p* = 0.024; and *CCNE1*, HR = 13.07 (95% CI, 2.47–69.24), *p* = 0.003] (Figure 5A). T stage was the only independent risk factor for OS [HR = 3.58 (95% CI, 1.61–7.98), *p* = 0.002]. Even *CCNE1* amplification was the independent risk factor for DFS in any patient with TNBC [HR = 13.48 (95% CI, 2.62–69.23), *p* = 0.002] (Figure 5B).

### *CCNE1* Amplification in Public Databases

*CCNE1* amplification was more frequently detected in TNBCs (10%, 21/209 in METABRIC and 13%, 16/119 in TCGA) compared to non-TNBCs (1.9%, 34/1,771 in METABRIC, *p* < 0.0001, Supplementary Figure 3A; and 2.9%, 28/961 in TCGA, *p* < 0.0001, Supplementary Figure 3B). In addition, the distinction translated into significant OS differences that TNBC with amplified *CCNE1* was associated with worse overall survival (*p* = 0.016, Supplementary Figure 3C) in METABRIC.

### IHC Confirmed Strong Intensity of Cyclin E1 in TNBC With *CCNE1* Amplification

**Figure 6:**
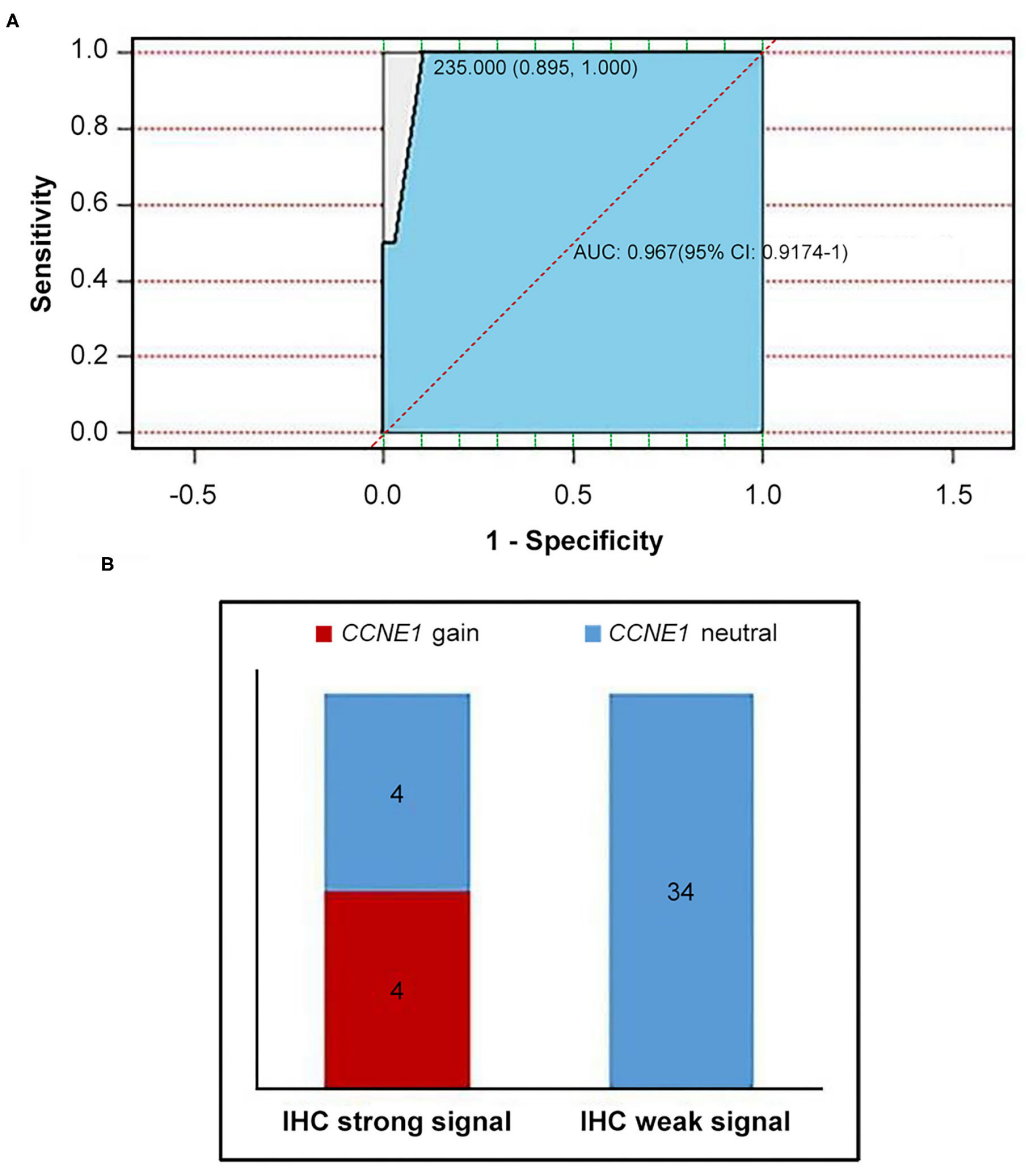
Consistency between overexpression of cyclin E1 and *CCNE1* amplification was showed. **(A)** AUC value confirmed consistency between *CCNE1* amplification and cyclin E1 protein expression. **(B)** Histogram for two groups divided by cutoff value of 235 (IHC strong signal and IHC weak signal). IHC, immunohistochemistry.

IHC staining for cyclin E1 was performed in 42 non-*BRCA* carriers with TNBC, including four patients with *CCNE1* amplification. A final consensus score for cyclin E1 staining by IHC was obtained by multiplying the intensity and extension values to achieve a score ranging from 0 to 300. As shown by the ROC curve, there was a good consistency between the IHC staining for cyclin E1 and the somatic mutation status of *CCNE1*, with an AUC of 0.967 (95% CI, 0.9174–1, Figure 6A). Based on a cutoff cyclin E1 consensus score of 235, 42 non-*BRCA* carriers were divided into two groups, those with strong and weak cyclin E1 signals. All four patients with *CCNE1* amplification were in the strong cyclin E1 signal group (Figure 6B), and the strong signal intensity of cyclin E1 was confirmed for all four patients with *CCNE1* amplification (Supplementary Figure 4). Based on the IHC staining for cyclin E1, strong cyclin E1 signal intensity by IHC tended to be associated with worse DFS (Supplementary Figure 5).

## Discussion

In contrast to the previous comprehensive mutational spectrum in TNBC (31, 34, 47), we performed an NGS-based analysis by comparing 21 germline *BRCA* carriers and 54 non-*BRCA* carriers in Chinese patients with TNBC. To the best of our knowledge, our study is the first to elucidate a more comprehensive comparison that included clinicopathological characteristics, genomic profiles, immunity-associated parameters and survival analysis. In addition, based on the eligibility criteria including a tumor size of ≥2 cm and/or ≥1 affected lymph nodes, we described the genomic profiles of TNBC cases with high tumor burden and worse prognosis compared to comprehensive unselected TNBC (48). The genomic profiles in this study was rare (49). In the current cohort, 14 of the 54 non-*BRCA* carriers (25.9%) and four of the 21 *BRCA* carriers (19.0%) died even though the follow-up was not long.

The prevalence of *BRCA1/2* mutations in the current cohort of TNBC cases reached 24.1%, which was close to the reported rate of 21.4% in unselected Chinese populations (50), indicating that *BRCA1/2* mutations were not related to high tumor burden or worse prognosis, in agreement with the findings of the POSH study (16). Our analysis revealed a mutation rate of 11.11% (6/54) in other cancer predisposition genes beyond *BRCA1/2* in non-*BRCA* carriers, which was only slightly lower than that in the germline *BRCA1/2* mutation carriers [14.19% (21/148)] in TNBC. Therefore, in addition to *BRCA1/2*, we should not overlook the clinical value of other germline mutation tests in TNBC patients in China. Among other germline mutations, one (1.85%, 1/54) mismatch repair gene mutation (*MSH3*) was found, which was parallel to that reported in a previous study (51), although the correlation between the germline *MSH3* mutation and TNBC remains to be explored (52). Except for *MSH3*, the rest of the germline mutant genes in non-*BRCA* carriers with TNBC were involved in the HRR pathway, including *BLM, PALB2, NBN, RAD51C*, and *RAD51D*, with the mutation rate of 9.26% (5/54), which increased the risk of other cancers, such as *PLAB2* for pancreatic cancer (53). The germline mutation rate of the members of the Fanconi anemia gene family (including *BRCA2, PALB2*, and *RAD51C*) was 25.93% (7/27) among patients with germline mutations in TNBC, which was lower than those in unselected breast cancer (35). These data strongly support the inclusion of not only *BRCA1/2* but also other germline mutations, such as HRR pathway genes, Lynch Syndrome, and Fanconi anemia (54), in the assessment of germline mutations in patients with TNBC.

In addition to *BRCA1/2*, growing evidence indicates that other germline mutations involved in HRR pathway genes may improve sensitivity to therapeutic agents such as platinum-based chemotherapy (55) and PARP inhibitors (56), implying that a few non-*BRCA* carriers with TNBC with other accompanying germline mutations involved in HRR pathway genes may benefit from platinum-based chemotherapy or PARP inhibitors.

Furthermore, our study has identified distinct somatic mutations among germline *BRCA* and non-*BRCA* carriers. *GRM3* mutation was detected more in *BRCA* carriers, while somatic *PIK3CA* missense was only detected in non-*BRCA* carriers. *GRM3* was reported to be the only genetic marker associated with bipolar disorder (57). However, the relationship between germline *BRCA* mutation and somatic *GRM3* alteration remains to be explored. Alpelisib has been approved for the treatment of advanced breast cancer with *PIK3CA* mutation in positive hormone receptor (58), and clinical trials are investigating the possible response of TNBC with *PIK3CA* mutation to alpelisib (59).

We specifically investigated the association between somatic mutations involved in HRR pathway genes and germline *BRCA1/2* status and found coexistence between germline *BRCA1* mutation and somatic *PLAB2* pathogenic variation in one patient. Although it did not directly conform to Knudson's “two-hit” paradigm (60), *PALB2* may be an example of a cancer predisposition gene (61).

Although a significant portion (22.22%, 12/54) of non-*BRCA* carriers also carry other somatic mutations involving HRR pathway genes, platinum-based chemotherapy, or PARP inhibitors may have no response in these cases as missense dominates the mutation type and this type might not result in homologous recombination defect (33, 62).

PD-1/PD-L1 blockade has been shown to have an acceptable safety profile and antitumor activity for TNBC in the phase Ib KEYNOTE-012 study (63), suggesting that ICIs are a promising therapeutic approach for TNBC cases with accumulated mutations. We aimed to investigate whether a high TMB and MSI-H, which are considered as predictive markers for survival after immunotherapy in other tumors (64–66), were also suitable as predictors in patients with TNBC. The median TMB, which was 4.1 Muts/Mb in both the *BRCA* and non-*BRCA* carriers in the present study, was very similar to the median TMB of 3.8 Muts/Mb in patients with breast cancer reported by Chalmers et al. (67); even lower than Japanese population reported by Nagahashi et al. (68) which was 11.5 (3.9–56.2)/Mb; far lower than TMB in melanoma (65) and non-small cell lung cancer (66) which was at least higher than 10 Muts/Mb. Thus, a high TMB was rare in TNBC. Furthermore, MSI-H was absent in this study. MSI-H was reported uncommon (0.9%) in TNBCs for Japanese population (69). Thus, we presume that neither a high TMB alone nor MSI-H alone is a suitable predictive marker for immunomodulation in TNBC because they do not represent the comprehensive immune environment in TNBC. These findings suggest that a combination of ICIs and other therapies should be considered as treatment approaches for TNBC. Several clinical trials are testing specific combinations of ICIs and PARP inhibitors such as olaparib, niraparib, and talazoparib, with preliminary data indicating their efficacy (70, 71).

There is an urgent need to discover new potential predictors and therapeutic targets for patients with TNBC, particularly for those whose prognosis is poor and who have poor response to chemotherapy (17) and no response to PARP inhibitors (20). Patients with high tumor burden, such as those with large tumor size (≥2 cm) and/or a higher proportion of affected lymph nodes (at least one affected lymph node) may have worse prognosis. Meanwhile, those without germline *BRCA* mutation may not respond to PARP inhibitors and may have poor response to platinum-based chemotherapy.

Our comparison of genomic profiling in *BRCA* and non-*BRCA* carriers led us to the unexpected discovery that *CCNE1* amplification was only detected in non-*BRCA* carriers. By comparing the survival of non-*BRCA* carriers with TNBC based on *CCNE1* status, those with *CCNE1* amplification showed worse DFS and a tendency of worse OS. Therefore, we focused our attention on *CCNE1* and were surprised to find that *CCNE1* amplification was an independent risk factor in non-*BRCA* carriers with TNBC even as an independent risk factor in TNBC.

Available large-scale public genomic databases from The Cancer Genome Atlas (TCGA) project (44) and the Molecular Taxonomy of Breast Cancer International Consortium (METABRIC) (43) have significantly improved our understanding of breast cancer tumor biology. To strength the importance of *CCNE1* amplification for TNBC, we also found the significant difference of amplified *CCNE1* between TNBCs and non-TNBCs both in METABRIC and TCGA databases. Surprisingly, *CCNE1* amplification was also confirmed to be associated with worse OS in METABRIC which was first reported.

There are few studies on the association between *CCNE1* amplification and TNBC prognosis. Only Zhao et al. (72) reported that *CCNE1* amplification may confer resistance to chemotherapy and is associated with poor overall survival in patients with TNBC. Although we drew the same conclusion, we went a step further that we confirmed amplified *CCNE1* was the independent risk factor for non-*BRCA* carriers with TNBC who really need attention due to being lack of distinct prognostic marker and therapeutic target. *CCNE1* amplification was mutually exclusive to germline *BRCA1/2* mutation which has been previously reported (35, 72). We determined its predictor role in the sub-group of TNBC, i.e., non-*BRCA* carriers with fewer options for treatment, such as PARP inhibitors. To translate and facilitate the application from DNA to protein level, IHC was used to confirm the consistency of *CCNE1* amplification and cyclin E1 overexpression in TNBC samples. With a good sensitivity of 100% and specificity of 89.5%, cyclin E1 IHC staining may have great potential for use instead of *CCNE1* amplification testing to facilitate routine clinical application on the basis that worse tendency of overall survival was still associated with strong intensity of Cyclin E1 according to IHC staining of Cyclin E1. This is the first to translate *CCNE1* at DNA level to Cyclin E1 at protein level.

Cyclin E1, as encoded by *CCNE1*, is the key kinase complex for cell cycle regulation from G1 to S phase. *CCNE1* amplification has also been observed in some other tumors (73, 74) and may lead to continuously activated DNA and centrosome replications, inducing chromosomal instability and tumor growth. Although cyclin E1-specific pharmacological inhibitors are not yet available, preclinical investigations as well as trials indirectly targeting *CCNE1* are both underway. Dariush Etemadmoghadam (75) showed the specific sensitivity of proteasome inhibitor bortezomib to *CCNE1-*amplified tumor cells. Furthermore, results of a study that applied proteasome inhibitor bortezomib as first-line therapy against multiple myeloma (76) indicate that it could also be considered as a potential therapeutic approach for TNBC with *CCNE1* amplification.

There are three limitations of our study. First, all 75 patients were from a single hospital. Second, the sample size for germline *BRCA1/2* mutation subgroup was <30 and certain mutation subtypes were very limited. Third, not all 75 patients had been followed up for more than 5 years. Therefore, future studies using larger sample sizes and long-term follow-up procedures should be conducted to investigate the correlation between specific mutations and survival outcomes.

## Conclusion

We explored intertumoral heterogeneity by comparing the differences in genomic profiles and immunity-associated parameters between germline *BRCA* and non-*BRCA* carriers in TNBC with high tumor burden. We revealed that both a high TMB and MSI-H were rare in patients with TNBC, indicating that these would not act as suitable predictors for TNBC for immune checkpoint inhibitors. Most notably, we discovered that amplified *CCNE1* may be a novel potential prognostic marker and therapeutic target for TNBC without germline *BRCA1/2* mutations. Overexpression of cyclin E1 may become a replacement for *CCNE1* amplification, which will facilitate its clinical application.

## Data Availability Statement

The dataset have been deposited to: http://db.cngb.org/ and the accession number is CNP0001304.

## Ethics Statement

The studies involving human participants were reviewed and approved by The study was approved by the Ethics Committee of Peking Union Medical College Hospital (No. HS-1623). The patients/participants provided their written informed consent to participate in this study.

## Author Contributions

QS contributed to conceptualization and design of the study. XH, DS, CZ, and CC organized the database. XH acquired funding. XH, DS, YZ, YL, and CW contributed the patients' resources. QS, XH, DS, DG, CZ, and BZ performed statistical analysis, immunochemistry, and NGS data analysis. XH, DS, YZ, YL, and TL investigated the patients. XH and DS wrote the first draft of the manuscript. All authors contributed to manuscript revision, read, and approved the submitted version.

## Conflict of Interest

DS and CZ were employed by the company BGI Genomics, Shenzhen, China. The remaining authors declare that the research was conducted in the absence of any commercial or financial relationships that could be construed as a potential conflict of interest.

## Supplementary Material

Supplementary Figure 1 Flow diagram of the patients in the study and analyses.

Supplementary Figure 2, related to Figure 1 and Table 2 Somatic alterations in the genomes of 75 Chinese patients with TNBC and germline *BRCA1/2* mutations in 21 Chinese patients with TNBC. **(A)** Percentages of types of somatic mutations. **(B)** Numbers of somatic mutations and germline *BRCA* mutations in individual patients. The numbers of genes with CNV loss per patient ranged from 0 to 3, whereas the numbers of amplified genes per patient ranged from 0 to 10. CNV, copy number variation; TNBC, triple-negative breast cancer.

Supplementary Figure 3 TNBCs exhibited more frequency with amplified *CCNE1* compared with non-TNBCs (Fisher's exact test) in **(A)** METABRIC: TNBC (*n* = 209) and non-TNBC (*n* = 1,771) (*p* < 0.0001) and in **(B**) TCGA: TNBC (*n* = 119) and non-TNBC (*n* = 961) (*p* < 0.0001). While TNBC with amplified *CCNE1* showed worse overall survival in METABRIC database: **(C)** Kaplan–Meier curve of OS with TNBC according to *CCNE1* CNV status in METABRIC. AMP, amplification; OS, overall survival; TNBC, triple-negative breast cancer.

Supplementary Figure 4, related to Figure 6 High-intensity levels of Cyclin E1 correlated positively with TNBC and *CCNE1* amplification under both low (40×) and high (100×) magnification in 4 patients in this study cohort. **(A)** Patient No. 56 **(B)** Patient No. 26 **(C)** Patient No. 28 **(D)** Patient No. 64.

Supplementary Figure 5 Kaplan–Meier analysis showed disease-free survival in non-*BRCA* carriers with TNBC according to IHC staining of Cyclin E1. IHC, immunohistochemistry.

Supplementary Table 1, related to Table 1 Clinicopathological characteristics of Chinese female patients with triple-negative breast cancer according to *BRCA* germline mutation status in this study cohort (p ≥ 0.05).

Supplementary Table 2 Events in *BRCA1/2* mutation carriers and non-*BRCA* carriers.

Supplementary Table 3, related to Table 3 Comparison of somatic mutations between *BRCA* germline mutation carriers and non-carriers of triple-negative breast cancer (mutation frequency equal to or more than 4% in the whole cohort, p ≥ 0.05).

Supplementary Table 4, related to Table 4 Comparison of somatic mutant genes involved in the homologous recombination repair pathway between *BRCA* germline mutation carriers and non-carriers of triple-negative breast cancer (p ≥ 0.05).

Supplementary Table 5 Univariate analysis of correlations between clinicopathological factors and genomic alterations and disease-free survival in non-*BRCA* carriers of triple-negative breast cancer.

Supplementary Table 6 Univariate analysis of correlations between clinicopathological factors and genomic alterations and overall survival in non-*BRCA* carriers of triple-negative breast cancer.

## Abbreviations

NGS: Next-generation sequencing
TNBC: Triple-negative breast cancer
LVI: Lymphovascular invasion
PUMCH: Peking Union Medical College Hospital
ER: Estrogen receptor
PR: Progesterone receptor
DFS: Disease-free survival
OS: Overall survival
HRR: Homologous recombination repair
PARP: Poly-ADP-ribose polymerase
CNV: Copy number variations
TMB: Tumor mutation burden
MSI: Microsatellite instability
METABRIC: Molecular Taxonomy of Breast Cancer International Consortium
TCGA: The Cancer Genome Atlas
FFPE: Formalin-fixed and paraffin-embedded
IHC: Immunohistochemistry
AUC: Area under the curve.

## References

[1] Dent R Trudeau M Pritchard KI Hanna WM Kahn HK Sawka CA. Triple-negative breast cancer: clinical features and patterns of recurrence. Clin Cancer Res. (2007) 13(15 Pt 1):4429–34. 10.1158/1078-0432.CCR-06-3045 17671126

[2] Chacon RD Costanzo MV. Triple-negative breast cancer. Breast Cancer Res. (2010) 12(Suppl.)2:S3. 10.1186/bcr2574 21050424 PMC2972557

[3] Carey L Winer E Viale G Cameron D Gianni L Triple-negative breast cancer: disease entity or title of convenience? Nat Rev Clin Oncol. (2010) 7:683–92. 10.1038/nrclinonc.2010.154 20877296

[4] Dignam JJ Dukic V Anderson SJ Mamounas EP Wickerham DL Wolmark N. Hazard of recurrence and adjuvant treatment effects over time in lymph node-negative breast cancer. Breast Cancer Res Treat. (2009) 116:595–602. 10.1007/s10549-008-0200-5 18830816 PMC2711214

[5] Tung N Lin NU Kidd J Allen BA Singh N Wenstrup RJ. Frequency of germline mutations in 25 cancer susceptibility genes in a sequential series of patients with breast cancer. J Clin Oncol. (2016) 34:1460–8. 10.1200/JCO.2015.65.0747 26976419 PMC4872307

[6] Miki Y Swensen J Shattuck-Eidens D Futreal PA Harshman K Tavtigian S A strong candidate for the breast and ovarian cancer susceptibility gene BRCA1. Science. (1994) 266:66–71. 10.1126/science.7545954 7545954

[7] Wooster R Bignell G Lancaster J Swift S Seal S Mangion J. Identification of the breast cancer susceptibility gene BRCA2. Nature. (1995) 378:789–92. 10.1038/378789a0 8524414

[8] Zhang K Zhou J Zhu X Luo M Xu C Yu J Germline mutations of PALB2 gene in a sequential series of Chinese patients with breast cancer. Breast Cancer Res Treat. (2017) 166:865–73. 10.1007/s10549-017-4425-z 28825143

[9] Han MR Zheng W Cai Q Gao YT Zheng Y Bolla MK Evaluating genetic variants associated with breast cancer risk in high and moderate-penetrance genes in Asians. Carcinogenesis. (2017) 38:511–8. 10.1093/carcin/bgx010 28419251 PMC5963497

[10] Hollestelle A Wasielewski M Martens JW Schutte M Discovering moderate-risk breast cancer susceptibility genes. Curr Opin Genet Dev. (2010) 20:268–76. 10.1016/j.gde.2010.02.009 20346647

[11] Childers CP Childers KK Maggard-Gibbons M Macinko J National estimates of genetic testing in women with a history of breast or ovarian cancer. J Clin Oncol. (2017) 35:3800–6. 10.1200/JCO.2017.73.6314 28820644 PMC5707208

[12] Taylor A Brady AF Frayling IM Hanson H Tischkowitz M Turnbull C. Consensus for genes to be included on cancer panel tests offered by UK genetics services: guidelines of the UK Cancer Genetics Group. J Med Genet. (2018) 55:372–7. 10.1136/jmedgenet-2017-105188 29661970 PMC5992364

[13] Slavin TP Niell-Swiller M Solomon I Nehoray B Rybak C Blazer KR Clinical application of multigene panels: challenges of next-generation counseling and cancer risk management. Front Oncol. (2015) 5:208 10.3389/fonc.2015.00208 26484312 PMC4586434

[14] Zhang G Wang Y Chen B Guo L Cao L Ren C. Characterization of frequently mutated cancer genes in Chinese breast tumors: a comparison of Chinese and TCGA cohorts. Ann Transl Med. (2019) 7:179. 10.21037/atm.2019.04.23 31168460 PMC6526269

[15] Metzger-Filho O Tutt A de Azambuja E Saini KS Viale G Loi S. Dissecting the heterogeneity of triple-negative breast cancer. J Clin Oncol. (2012) 30:1879–87. 10.1200/JCO.2011.38.2010 22454417

[16] Copson ER Maishman TC Tapper WJ Cutress RI Greville-Heygate S Altman DG. Germline BRCA mutation and outcome in young-onset breast cancer (POSH): a prospective cohort study. Lancet Oncol. (2018) 19:169–80. 10.1016/S1470-2045(17)30891-4 29337092 PMC5805863

[17] Turner NC Tutt ANj. Platinum chemotherapy for BRCA1-related breast cancer: do we need more evidence? Breast Cancer Res. (2012) 14:115. 10.1186/bcr3332 23146216 PMC4053124

[18] Byrski T Gronwald J Huzarski T Grzybowska E Budryk M Stawicka M. Pathologic complete response rates in young women with BRCA1-positive breast cancers after neoadjuvant chemotherapy. J Clin Oncol. (2010) 28:375–9. 10.1200/JCO.2008.20.7019 20008645

[19] Jiang T Shi W Wali VB Pongor LS Li C Lau R. Predictors of chemosensitivity in triple negative breast cancer: an integrated genomic analysis. PLoS Med. (2016) 13:e1002193. 10.1371/journal.pmed.1002193 27959926 PMC5154510

[20] Robson M Ruddy KJ Im SA Senkus E Xu B Domchek SM. Patient-reported outcomes in patients with a germline BRCA mutation and HER2-negative metastatic breast cancer receiving olaparib versus chemotherapy in the OlympiAD trial. Eur J Cancer. (2019) 120:20–30. 10.1016/j.ejca.2019.06.023 31446213 PMC6836724

[21] Kurian AW Ward KC Hamilton AS Deapen DM Abrahamse P Bondarenko I. Uptake, results, and outcomes of germline multiple-gene sequencing after diagnosis of breast cancer. JAMA Oncol. (2018) 4:1066–72. 10.1001/jamaoncol.2018.0644 29801090 PMC6143044

[22] Zhou M Zhong L Xu W Sun Y Zhang Z Zhao H. Discovery of potential prognostic long non-coding RNA biomarkers for predicting the risk of tumor recurrence of breast cancer patients. Sci Rep. (2016) 6:31038. 10.1038/srep31038 27503456 PMC4977495

[23] Sun J Chen X Wang Z Guo M Shi H Wang X. A potential prognostic long non-coding RNA signature to predict metastasis-free survival of breast cancer patients. Sci Rep. (2015) 5:16553. 10.1038/srep16553 26549855 PMC4637883

[24] Barroso-Sousa R Jain E Cohen O Kim D Buendia-Buendia J Winer E. Prevalence and mutational determinants of high tumor mutation burden in breast cancer. Ann Oncol. (2020) 31:387–94. 10.1016/j.annonc.2019.11.010 32067680

[25] Fusco N Lopez G Corti C Pesenti C Colapietro P Ercoli G. Mismatch repair protein loss as a prognostic and predictive biomarker in breast cancers regardless of microsatellite instability. JNCI Cancer Spectr. (2018) 2:pky056. 10.1093/jncics/pky056 31360876 PMC6649738

[26] Bertucci F Goncalves A. Immunotherapy in breast cancer: the emerging role of PD-1 and PD-L1. Curr Oncol Rep. (2017) 19:64. 10.1007/s11912-017-0627-0 28799073

[27] Barroso-Sousa R Keenan TE Pernas S Exman P Jain E Garrido-Castro AC. Tumor mutational burden and PTEN alterations as molecular correlates of response to PD-1/L1 blockade in metastatic triple-negative breast cancer. Clin Cancer Res. (2020) 26:2565–72. 10.1158/1078-0432.CCR-19-3507 32019858 PMC7269810

[28] Horimoto Y Thinzar Hlaing M Saeki H Kitano S Nakai K Sasaki R. Microsatellite instability and mismatch repair protein expressions in lymphocyte-predominant breast cancer. Cancer Sci. (2020) 111:2647–54. 10.1111/cas.14500 32449246 PMC7385389

[29] Sun J Zhang Z Bao S Yan C Hou P Wu N. Identification of tumor immune infiltration-associated lncRNAs for improving prognosis and immunotherapy response of patients with non-small cell lung cancer. J Immunother Cancer. (2020) 8:e000110.10.1136/jitc-2019-000110 10.1136/jitc-2019-000110 32041817 PMC7057423

[30] Bao S Zhao H Yuan J Fan D Zhang Z Su J. Computational identification of mutator-derived lncRNA signatures of genome instability for improving the clinical outcome of cancers: a case study in breast cancer. Brief Bioinform. (2019) 21:1742–55. 10.1093/bib/bbz118 31665214

[31] Jiang YZ Ma D Suo C Shi J Xue M Hu X. Genomic and transcriptomic landscape of triple-negative breast cancers: subtypes and treatment strategies. Cancer Cell. (2019) 35:428–40 e5. 10.1016/j.ccell.2019.02.001 30853353

[32] Shao F Sun H Deng CX. Potential therapeutic targets of triple-negative breast cancer based on its intrinsic subtype. Oncotarget. (2017) 8:73329–44. 10.18632/oncotarget.20274 29069872 PMC5641215

[33] Staaf J Glodzik D Bosch A Vallon-Christersson J Reutersward C Hakkinen J. Whole-genome sequencing of triple-negative breast cancers in a population-based clinical study. Nat Med. (2019) 25:1526–33. 10.1038/s41591-019-0582-4 31570822 PMC6859071

[34] Jeong HM Kim RN Kwon MJ Oh E Han J Lee SK. Targeted exome sequencing of Korean triple-negative breast cancer reveals homozygous deletions associated with poor prognosis of adjuvant chemotherapy-treated patients. Oncotarget. (2017) 8:61538–50. 10.18632/oncotarget.18618 28977883 PMC5617443

[35] Chen B Zhang G Li X Ren C Wang Y Li K. Comparison of BRCA versus non-BRCA germline mutations and associated somatic mutation profiles in patients with unselected breast cancer. Aging. (2020) 12:3140–55. 10.18632/aging.102783 32091409 PMC7066887

[36] Ding J Wu W Fang J Chu Y Zheng S Jiang L. Changes of breast cancer staging when AJCC prognostic staging manual is used: a retrospective analysis of a Chinese cohort. Int J Biol Markers. (2018) 33:168–73. 10.5301/ijbm.5000302 28967067

[37] Wolff AC Hammond MEH Allison KH Harvey BE Mangu PB Bartlett JMS. Human epidermal growth factor receptor 2 testing in breast cancer: American Society of Clinical Oncology/College of American pathologists clinical practice guideline focused update. J Clin Oncol. (2018) 36:2105–22. 10.1200/JCO.2018.77.8738 29846122

[38] Guan Y Hu H Peng Y Gong Y Yi Y Shao L. Detection of inherited mutations for hereditary cancer using target enrichment and next generation sequencing. Fam Cancer. (2015) 14:9–18. 10.1007/s10689-014-9749-9 25151137

[39] Salipante SJ Scroggins SM Hampel HL Turner EH Pritchard CC. Microsatellite instability detection by next generation sequencing. Clin Chem. (2014) 60:1192–9. 10.1373/clinchem.2014.223677 24987110

[40] Niu B Ye K Zhang Q Lu C Xie M McLellan MD. MSIsensor: microsatellite instability detection using paired tumor-normal sequence data. Bioinformatics. (2014) 30:1015–6. 10.1093/bioinformatics/btt755 24371154 PMC3967115

[41] Kautto EA Bonneville R Miya J Yu L Krook MA Reeser JW. Performance evaluation for rapid detection of pan-cancer microsatellite instability with MANTIS. Oncotarget. (2017). 8:7452–63. 10.18632/oncotarget.13918 27980218 PMC5352334

[42] Liang Z Zhang J Zeng X Gao J Wu S Liu T. Relationship between EGFR expression, copy number and mutation in lung adenocarcinomas. BMC Cancer. (2010) 10:376. 10.1186/1471-2407-10-376 20637128 PMC2913962

[43] Curtis C Shah SP Chin SF Turashvili G Rueda OM Dunning MJ. The genomic and transcriptomic architecture of 2,000 breast tumours reveals novel subgroups. Nature. (2012) 486:346–52. 10.1038/nature10983 22522925 PMC3440846

[44] Cancer Genome Atlas Network. Comprehensive molecular portraits of human breast tumours. Nature. (2012) 490:61–70. 10.1038/nature11412 23000897 PMC3465532

[45] Gao J Aksoy BA Dogrusoz U Dresdner G Gross B Sumer SO. Integrative analysis of complex cancer genomics and clinical profiles using the cBioPortal. Sci Signal. (2013) 6:pl1. 10.1126/scisignal.2004088 23550210 PMC4160307

[46] Li J Lupat R Amarasinghe KC Thompson ER Doyle MA Ryland GL. CONTRA: copy number analysis for targeted resequencing. Bioinformatics. (2012) 28:1307–13. 10.1093/bioinformatics/bts146 22474122 PMC3348560

[47] Shah SP Roth A Goya R Oloumi A Ha G Zhao Y. The clonal and mutational evolution spectrum of primary triple-negative breast cancers. Nature. (2012) 486:395–9. 10.1038/nature10933 22495314 PMC3863681

[48] Chandra D Suresh P Sinha R Azam S Batra U Talwar V. Eight year survival analysis of patients with triple negative breast cancer in India. Asian Pac J Cancer Prev. (2016) 17:2995–9. 27356724

[49] Jiang G Zhang S Yazdanparast A Li M Pawar AV Liu Y. Comprehensive comparison of molecular portraits between cell lines and tumors in breast cancer. BMC Genomics. (2016) 17 (Suppl. 7):525. 10.1186/s12864-016-2911-z 27556158 PMC5001206

[50] Zhong X Dong Z Dong H Li J Peng Z Deng L. Prevalence and prognostic role of BRCA1/2 variants in unselected Chinese breast cancer patients. PLoS ONE. (2016) 11:e0156789. 10.1371/journal.pone.0156789 27257965 PMC4892623

[51] Lee E Levine EA Franco VI Allen GO Gong F Zhang Y. Combined genetic and nutritional risk models of triple negative breast cancer. Nutr Cancer. (2014) 66:955–63. 10.1080/01635581.2014.932397 25023197

[52] Mills AM Dill EA Moskaluk CA Dziegielewski J Bullock TN Dillon P.M.. The relationship between mismatch repair deficiency and PD-L1 expression in breast Carcinoma. Am J Surg Pathol. (2018) 42:183–91. 10.1097/PAS.0000000000000949 28914717

[53] Tischkowitz M Xia B. PALB2/FANCN: recombining cancer and Fanconi anemia. Cancer Res. (2010) 70:7353–9. 10.1158/0008-5472.CAN-10-1012 20858716 PMC2948578

[54] Nikolaidis C Ming C Pedrazzani C van der Horst T Kaiser-Grolimund A Ademi Z. Challenges and opportunities for cancer predisposition cascade screening for hereditary breast and ovarian cancer and lynch syndrome in Switzerland: findings from an International Workshop. Public Health Genomics. (2018) 21:121–32. 10.1159/000496495 30695780

[55] Wattenberg MM Asch D Yu S O'Dwyer PJ Domchek SM Nathanson KL Platinum response characteristics of patients with pancreatic ductal adenocarcinoma and a germline BRCA1, BRCA2 or PALB2 mutation. Br J Cancer. (2020) 122:333–9. 10.1038/s41416-019-0582-7 31787751 PMC7000723

[56] Telli ML Timms KM Reid J Hennessy B Mills GB Jensen KC. Homologous recombination deficiency (HRD) score predicts response to platinum-containing neoadjuvant chemotherapy in patients with triple-negative breast cancer. Clin Cancer Res. (2016) 22:3764–73. 10.1158/1078-0432.CCR-15-2477 26957554 PMC6773427

[57] Kandaswamy R McQuillin A Sharp SI Fiorentino A Anjorin A Blizard RA. Genetic association, mutation screening, and functional analysis of a kozak sequence variant in the metabotropic glutamate receptor 3 gene in bipolar disorder. JAMA Psychiatry. (2013) 70:591–8. 10.1001/jamapsychiatry.2013.38 23575746

[58] Andre F Ciruelos E Rubovszky G Campone M Loibl S Rugo HS. Alpelisib for PIK3CA-mutated, hormone receptor-positive advanced breast cancer. N Engl J Med. (2019) 380:1929–40. 10.1056/NEJMoa1813904 31091374

[59] Teo ZL Versaci S Dushyanthen S Caramia F Savas P Mintoff CP. Combined CDK4/6 and PI3Kalpha inhibition is synergistic and immunogenic in triple-negative breast cancer. Cancer Res. (2017) 77:6340–52. 10.1158/0008-5472.CAN-17-2210 28947417

[60] Hino O Kobayashi T. Mourning Dr. Alfred G. Knudson: the two-hit hypothesis, tumor suppressor genes, and the tuberous sclerosis complex. Cancer Sci. (2017) 108:5–11. 10.1111/cas.13116 27862655 PMC5276834

[61] Lee JEA Li N Rowley SM Cheasley D Zethoven M McInerny S. Molecular analysis of PALB2-associated breast cancers. J Pathol. (2018) 245:53–60.10.1002/path.5055 10.1002/path.5055 29431189

[62] Lu J Wu D Li C Zhou M Hao D. Correlation between gene expression and mutator phenotype predicts homologous recombination deficiency and outcome in ovarian cancer. J Mol Med. (2014) 92:1159–68. 10.1007/s00109-014-1191-9 25062964

[63] Nanda R Chow LQ Dees EC Berger R Gupta S Geva R. Pembrolizumab in patients with advanced triple-negative breast cancer: phase Ib KEYNOTE-012 study. J Clin Oncol. (2016) 34:2460–7. 10.1200/JCO.2015.64.8931 27138582 PMC6816000

[64] Schmid P Rugo HS Adams S Schneeweiss A Barrios CH Iwata H. Atezolizumab plus nab-paclitaxel as first-line treatment for unresectable, locally advanced or metastatic triple-negative breast cancer (IMpassion130): updated efficacy results from a randomised, double-blind, placebo-controlled, phase 3 trial. Lancet Oncol. (2020) 21:44–59. 10.1016/S1470-2045(19)30689-8 31786121

[65] Chan TA Wolchok JD Snyder A. Genetic basis for clinical response to CTLA-4 blockade in melanoma. N Engl J Med. (2015) 373:1984. 10.1056/NEJMc1508163 26559592

[66] Rizvi NA Hellmann MD Snyder A Kvistborg P Makarov V Havel JJ. Cancer immunology, mutational landscape determines sensitivity to PD-1 blockade in non-small cell lung cancer. Science. (2015) 348:124–8. 10.1126/science.aaa1348 25765070 PMC4993154

[67] Chalmers ZR Connelly CF Fabrizio D Gay L Ali SM Ennis R. Analysis of 100,000 human cancer genomes reveals the landscape of tumor mutational burden. Genome Med. (2017) 9:34. 10.1186/s13073-017-0424-2 28420421 PMC5395719

[68] Masayuki N Yiwei L Tetsu H Yuko K Manabu F Kazuhiro Y Tumor mutation burden in triple negative breast cancer patients in Japan. JCO. (2018) 36 (Suppl.):e13111 10.1200/JCO.2018.36.15_suppl.e13111

[69] Kurata K Kubo M Kai M Mori H Kawaji H Kaneshiro K. Microsatellite instability in Japanese female patients with triple-negative breast cancer. Breast Cancer. (2020) 27:490–8. 10.1007/s12282-019-01043-5 31907878 PMC7196096

[70] Mouw KW Goldberg MS Konstantinopoulos PA D'Andrea AD. DNA damage and repair biomarkers of immunotherapy response. Cancer Discov. (2017) 7:675–93. 10.1158/2159-8290.CD-17-0226 28630051 PMC5659200

[71] Vinayak S Tolaney SM Schwartzberg L Mita M McCann G Tan AR Open-label clinical trial of niraparib combined with pembrolizumab for treatment of advanced or metastatic triple-negative breast cancer. JAMA Oncol. (2019) 5:1132–40. 10.1001/jamaoncol.2019.1029 PMC6567845 31194225

[72] Zhao ZM Yost SE Hutchinson KE Li SM Yuan YC Noorbakhsh J. CCNE1 amplification is associated with poor prognosis in patients with triple negative breast cancer. BMC Cancer. (2019) 19:96. 10.1186/s12885-019-5290-4 30665374 PMC6341717

[73] Kawamura K Izumi H Ma Z Ikeda R Moriyama M Tanaka T. Induction of centrosome amplification and chromosome instability in human bladder cancer cells by p53 mutation and cyclin E overexpression. Cancer Res. (2004) 64:4800–9. 10.1158/0008-5472.CAN-03-3908 15256449

[74] Nakayama N Nakayama K Shamima Y Ishikawa M Katagiri A Iida K. Gene amplification CCNE1 is related to poor survival and potential therapeutic target in ovarian cancer. Cancer. (2010) 116:2621–34. 10.1002/cncr.24987 20336784

[75] Etemadmoghadam D Weir BA Au-Yeung G Alsop K Mitchell G George J. Synthetic lethality between CCNE1 amplification and loss of BRCA1. Proc Natl Acad Sci USA. (2013) 110:19489–94. 10.1073/pnas.1314302110 24218601 PMC3845173

[76] Scott K Hayden PJ Will A Wheatley K Coyne I. Bortezomib for the treatment of multiple myeloma. Cochrane Database Syst Rev. (2016) 4:CD010816. 10.1002/14651858.CD010816.pub2 27096326 PMC10387344

