# Supplementary figures and images for: Genomic Profiling Comparison of Germline BRCA and Non-BRCA Carriers Reveals CCNE1 Amplification as a Risk Factor for Non-BRCA Carriers in Patients With Triple-Negative Breast Cancer
Source: Front Oncol. 2020 Oct 30;10:583314. doi: 10.3389/fonc.2020.583314 (PMC7662137; doi:10.3389/fonc.2020.583314)

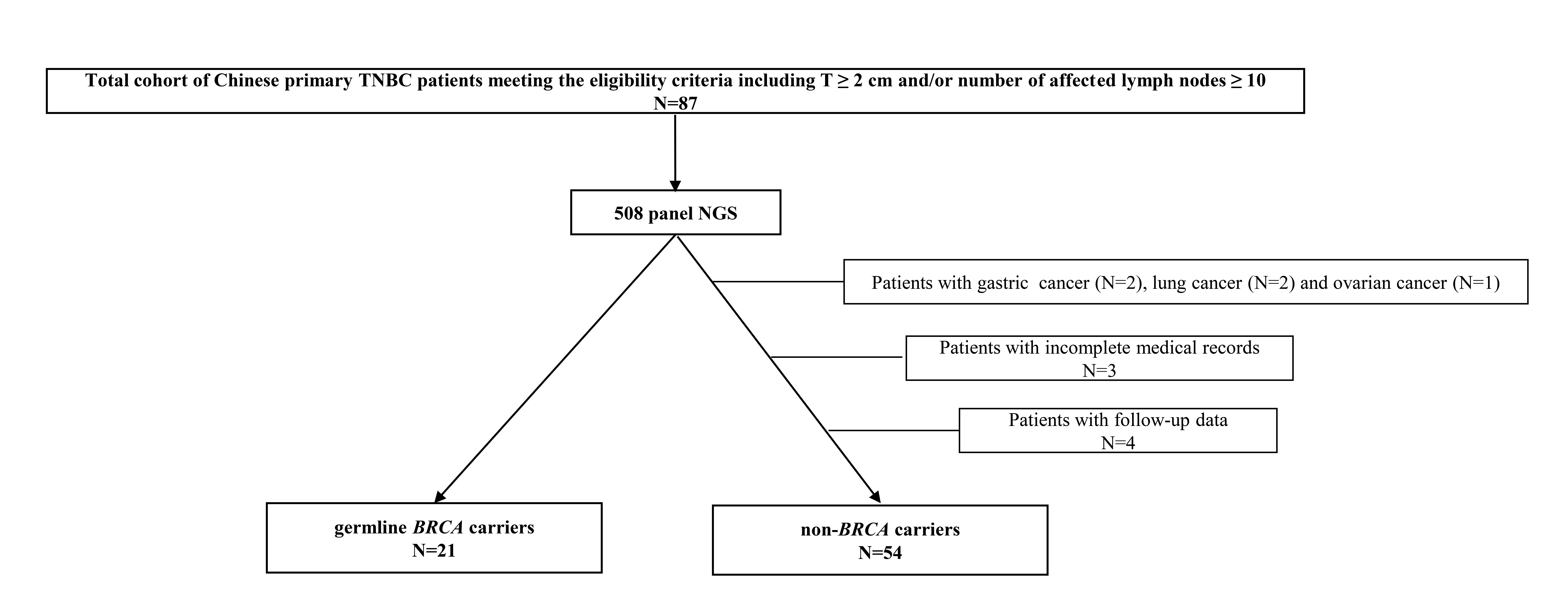

Supplement: Supplementary Figure 1 — Flow diagram of the patients in the study and analyses. [file Image_1.jpg]

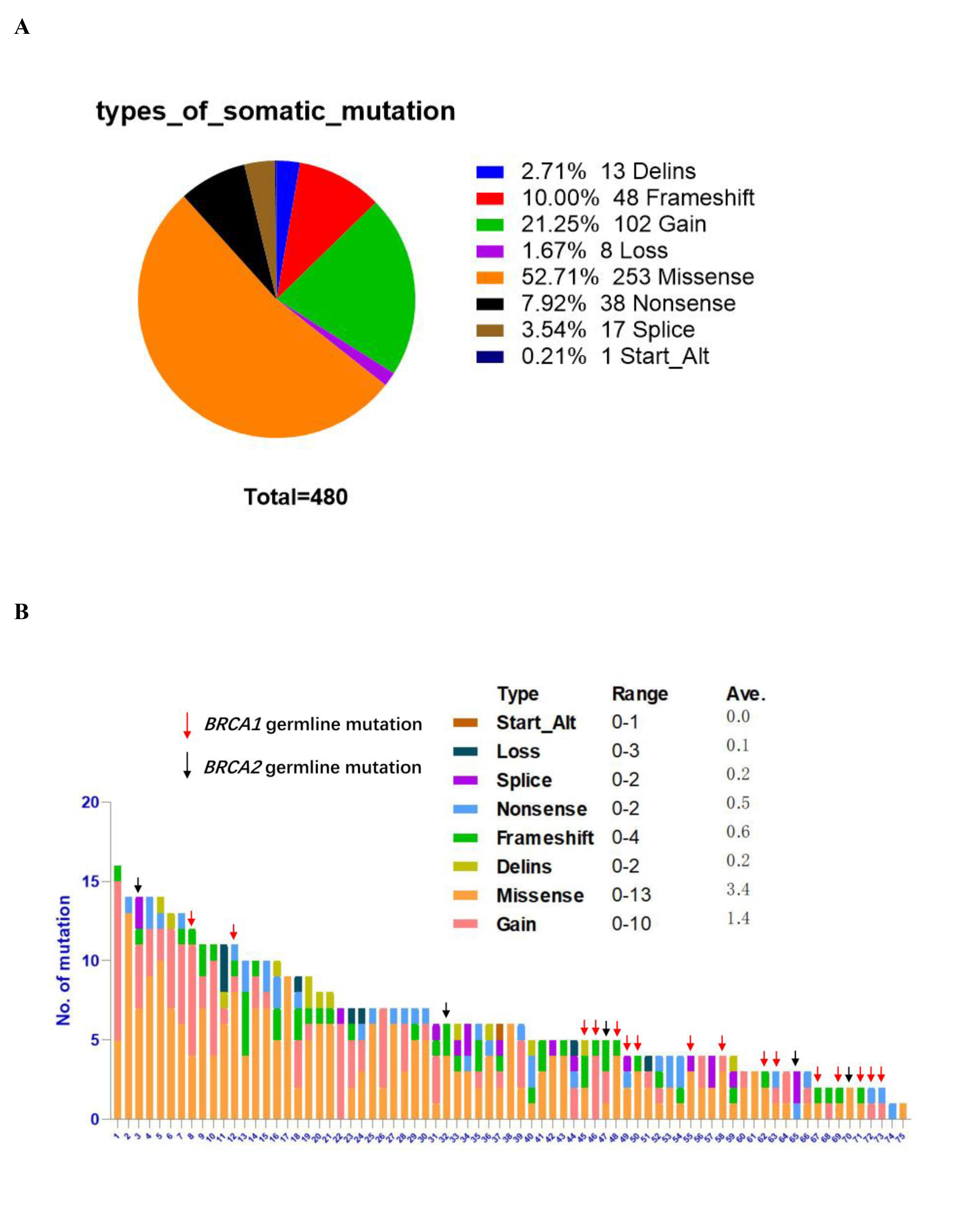

Supplement: Supplementary Figure 2, related to Figure 1 and Table 2 — Somatic alterations in the genomes of 75 Chinese patients with TNBC and germline BRCA1/2 mutations in 21 Chinese patients with TNBC. (A) Percentages of types of somatic mutations. (B) Numbers of somatic mutations and germline BRCA mutations in individual patients. The numbers of genes with CNV loss per patient ranged from 0 to 3, whereas the numbers of amplified genes per patient ranged from 0 to 10. CNV, copy number variation; TNBC, triple-negative breast cancer. [file Image_2.jpg]

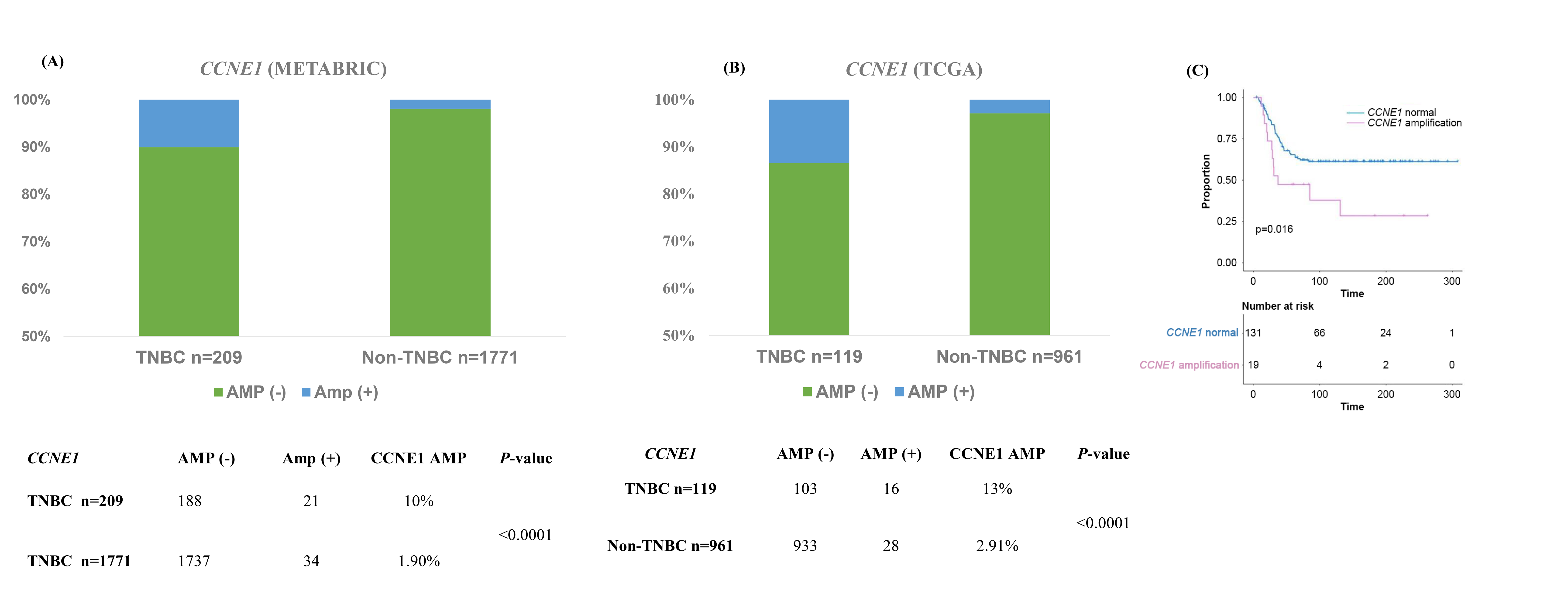

Supplement: Supplementary Figure 3 — TNBCs exhibited more frequency with amplified CCNE1 compared with non-TNBCs (Fisher's exact test) in (A) METABRIC: TNBC (n = 209) and non-TNBC (n = 1,771) (p < 0.0001) and in (B) TCGA: TNBC (n = 119) and non-TNBC (n = 961) (p < 0.0001). While TNBC with amplified CCNE1 showed worse overall survival in METABRIC database: (C) Kaplan–Meier curve of OS with TNBC according to CCNE1 CNV status in METABRIC. AMP, amplification; OS, overall survival; TNBC, triple-negative breast cancer. [file Image_3.tif]

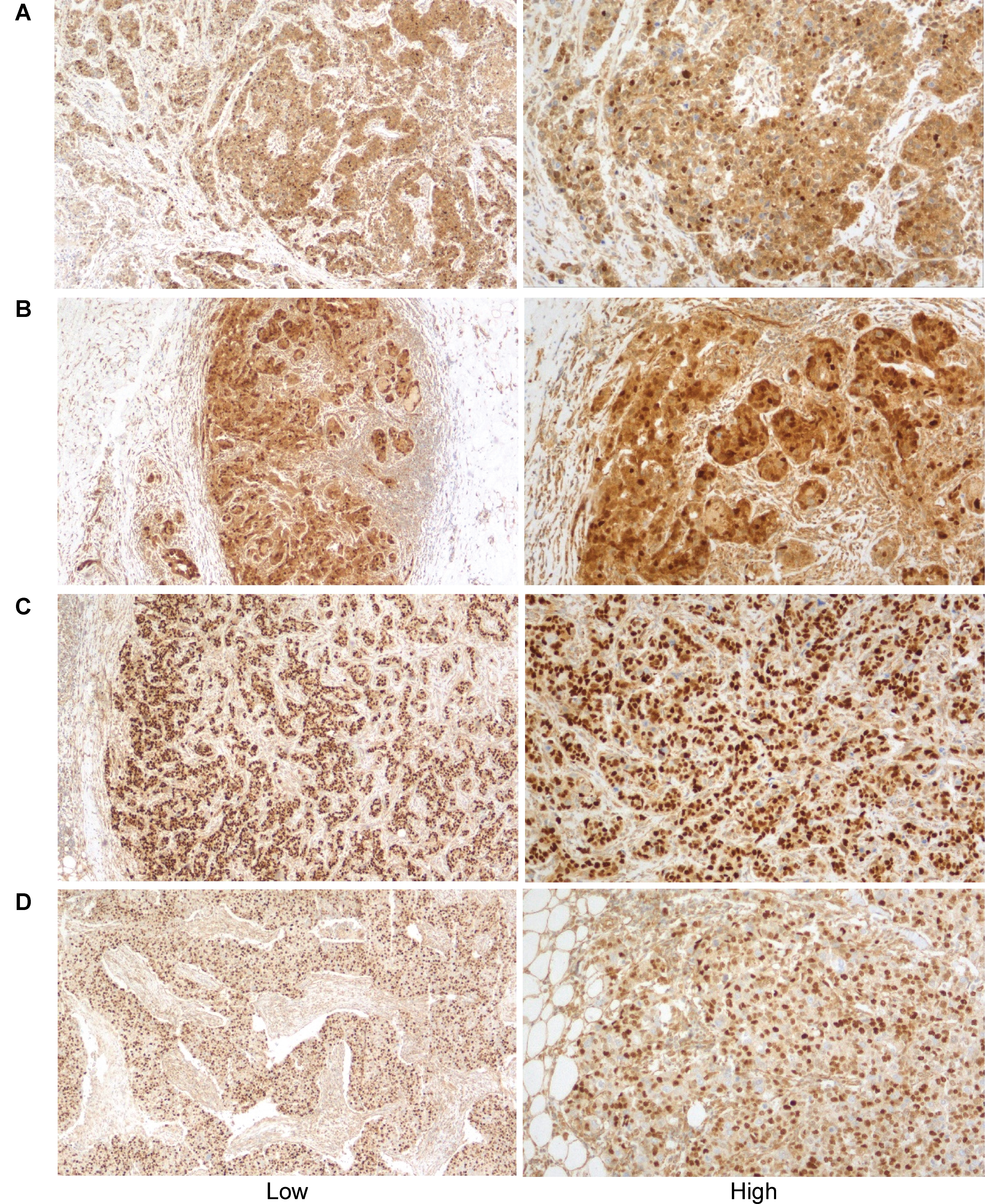

Supplement: Supplementary Figure 4, related to Figure 6 — High-intensity levels of Cyclin E1 correlated positively with TNBC and CCNE1 amplification under both low (40×) and high (100×) magnification in 4 patients in this study cohort. (A) Patient No. 56 (B) Patient No. 26 (C) Patient No. 28 (D) Patient No. 64. [file Image_4.jpg]

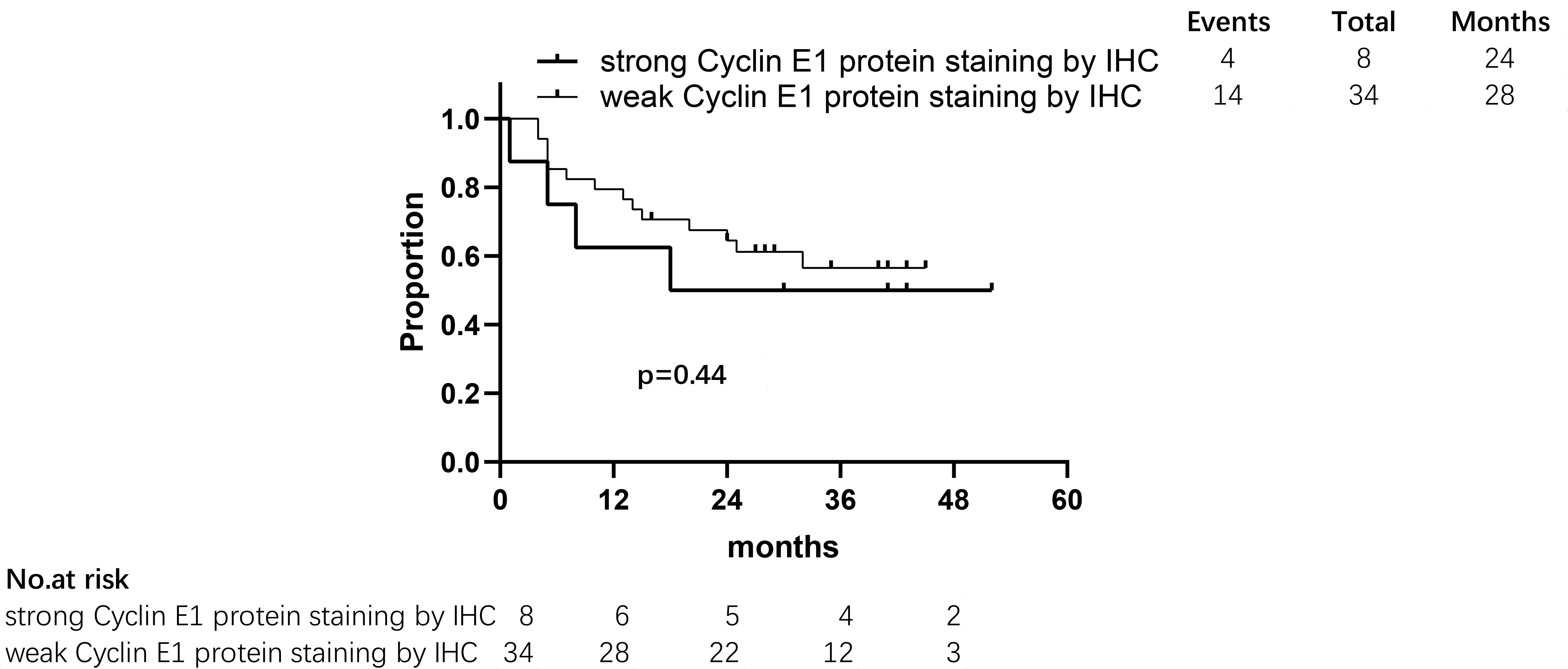

Supplement: Supplementary Figure 5 — Kaplan–Meier analysis showed disease-free survival in non-BRCA carriers with TNBC according to IHC staining of Cyclin E1. IHC, immunohistochemistry. [file Image_5.jpg]
