# Supplementary material for: Genomic Profiling Comparison of Germline BRCA and Non-BRCA Carriers Reveals CCNE1 Amplification as a Risk Factor for Non-BRCA Carriers in Patients With Triple-Negative Breast Cancer
Source: Front Oncol. 2020 Oct 30;10:583314. doi: 10.3389/fonc.2020.583314 (PMC7662137; doi:10.3389/fonc.2020.583314)
Supplement: Supplementary Table 1, related to Table 1 — Clinicopathological characteristics of Chinese female patients with triple-negative breast cancer according to BRCA germline mutation status in this study cohort (p ≥ 0.05). [file Table_1.DOCX]

**Table S1, related to Table 1. Clinicopathological characteristics of Chinese female patients with triple-negative breast cancer according to *BRCA* germline mutation status in this study cohort (p value ≥ 0.05)**

|  |  | **No. (%)** | | | | | | | | | | | | | |  |
| --- | --- | --- | --- | --- | --- | --- | --- | --- | --- | --- | --- | --- | --- | --- | --- | --- |
| **Characteristics (N=75)** | **Parameter** | ***BRCA* germline mutation** | | ***BRCA* non-carrier** | | | | | | | | ***p*-value** | | | | |
| Histology | IDC | 20 (95.2) | | 52 (96.3) | | | | | | | |  | | | | |
|  | other | 1 (4.8) | | 2 (3.7) | | | | | | | |  | | | | |
|  |  |  |  | | | |  | |  | | | | | | 0.83 | |
|  |  |  |  | | | |  | |  | | | | | |  | |
| T stage | 0 | 0 (0.0) | | 1 (1.9) | | | | | | | |  | | | | |
|  | 1 | 5 (23.8) | | 11 (20.4) | | | | | | | |  | | | | |
|  | 2 | 13 (61.9) | | 35 (64.8) | | | | | | | |  | | | | |
|  | 3 | 3 (14.3) | | | | 4 (7.4) | | | |  |  |  |  |  |  |  |
|  | 4 | 0 (0.0) | | 3 (5.5) | | | | | | | |  | | | | |
|  |  |  |  | | | |  | |  | | | | | | 0.65 | |
|  |  |  |  | | | |  | |  | | | | | |  | |
| TNM stage | I | 1 (4.8) | | 2 (3.7) | | | | | | | |  | | | | |
|  | II | 13 (61.9) | | 36 (66.7) | | | | | | | |  | | | | |
|  | III | 7 (33.3) | | 16 (29.6) | | | | | | | |  | | | | |
|  |  |  |  | | | |  | |  | | | | | | 0.99 | |
|  |  |  |  | | | |  | |  | | | | | |  | |
| LN status | Negative | 8 (38.1) | | 20 (37.1) | | | | | | | |  | | | | |
|  | Positive | 13 (61.9) | | 34 (62.9) | | | | | | | |  | | | | |
|  |  |  |  | | | |  | |  | | | | | | 0.93 | |
|  |  |  |  | | | |  | |  | | | | | |  | |
| Number of positive LN | 0 | 8 (38.1)  7 (33.3)  2 (9.5)  4 (19.1) | | | | 20 (37.0) | | | | |  |  |  |  |  |  |
|  | 1–3 |  |  |  |  | 17 (31.5) | | | | |  |  |  |  |  |  |
|  | 4–9 |  |  |  |  | 8 (14.8) | | | |  |  |  |  |  |  |  |
|  | >9 |  |  |  |  | 9 (16.7) | | | |  |  |  |  |  |  |  |
|  |  |  |  | | | |  | |  | | | | | | 0.94 | |
|  |  |  |  | | | |  | |  | | | | | |  | |
| Histological grade | Low grade | 0 (0.0)  1 (4.8)  20 (95.2) | | 1 (1.8)  13 (24.1)  40 (74.1) | | | | | | | |  | | | | |
|  | Medium grade |  |  |  |  |  |  |  |  |  |  |  | | | | |
|  | High grade |  |  |  |  |  |  |  |  |  |  |  | | | | |
|  |  |  |  | | | |  | |  | | | | | | 0.09 | |
|  |  |  |  | | | |  | |  | | | | | |  | |
| LVI | Negative | 18 (85.7)  3 (14.3) | | 42 (77.8)  12 (22.2) | | | | | | | |  | | | | |
|  | Positive |  |  |  |  |  |  |  |  |  |  |  | | | | |
|  |  |  |  | | | |  | |  | | | | | | 0.44 | |
|  |  |  |  | | | |  | |  | | | | | |  | |
| Ki-67 | <14% | 1 (4.8)  20 (95.2) | | 1 (1.8)  53 (98.2) | | | | | | | |  | | | | |
|  | ≥14% |  |  |  |  |  |  |  |  |  |  |  | | | | |
|  |  |  |  | | | |  | |  | | | | | | 0.48 | |
|  |  |  |  | | | |  | |  | | | | | |  | |
| CK5/6 | Negative | 12 (57.1)  9 (42.9) | | 28 (51.9)  26 (48.1) | | | | | | | |  | | | | |
|  | Positive |  |  |  |  |  |  |  |  |  |  |  | | | | |
|  |  |  |  | | | |  | |  | | | | | | 0.68 | |
|  |  |  |  | | | |  | |  | | | | | |  | |
| EGFR | Negative | 3 (14.3)  18 (85.7)  0 (0.0) | | 10 (18.5)  42 (77.8)  2 (3.7) | | | | | | | |  | | | | |
|  | Positive |  |  |  |  |  |  |  |  |  |  |  | | | | |
|  | Unknown |  |  |  |  |  |  |  |  |  |  |  | | | | |
|  |  |  |  | | | |  | |  | | | | | | 0.86 | |
|  |  |  |  | | | |  | |  | | | | | |  | |
| Basal-like | No | 1 (4.8)  20 (95.2) | | | | 3 (5.6) | | | | |  |  |  |  |  |  |
|  | Yes |  |  |  |  | 51 (94.4) | | | | |  |  |  |  |  |  |
|  |  |  |  | | | |  | |  | | | | | | 0.89 | |
|  |  |  |  | | | |  | |  | | | | | |  | |
|  |  |  |  | | | |  | |  | | | | | |  | |
| Surgical management of breast | Breast-conserving surgery | 5 (23.8) | | 12 (22.2) | | | | | | | |  | | | | |
|  | Mastectomy | 16 (76.2) | | 42 (77.8) | | | | | | | |  | | | | |
|  |  |  |  | | | |  | |  | | | | | | 0.88 | |
|  |  |  |  | | | |  | |  | | | | | |  | |
| Surgical management of axilla  (N=74) | ALND | 15 (71.4) | | 44 (81.5) | | | | | | | |  | | | | |
|  | SLN | 4 (19.1) | | 6 (11.1) | | | | | | | |  | | | | |
|  | SLN followed by ALND | 2 (9.5) | | 3 (5.5) | | | | | | | |  | | | | |
|  |  |  |  | | | |  | |  | | | | | | 0.53 | |
|  |  |  |  | | | |  | |  | | | | | |  | |
| Chemotherapy | Adjuvant chemotherapy | 20 (95.3) | | 52 (98.1) | | | | | | | |  | | | | |
| (N=74) | Neoadjuvant chemotherapy | 0 (0.0) | | 1 (1.9) | | | | | | | |  | | | | |
|  | Neoadjuvant + adjuvant chemotherapy | 1 (4.7) | | 0 (0.0) | | | | | | | |  | | | | |
|  |  |  |  | | | |  | |  | | | | | | 0.48 | |
|  |  |  |  | | | |  | |  | | | | | |  | |
| Chemotherapy cycle  (N=74) | ≤4 | 1 (4.8) | | | | | | 16 (30.2) | | | | |  | | | |
|  | 6 | 10 (47.6) | | | | | | 17 (32.1) | | | | |  | | | |
|  | 8 | 8 (38.1) | | | | | | 14 (26.4) | | | | |  | | | |
|  | ＞8 | 2 (9.5) | | | | | | 6 (11.3) | | | | |  | | | |
|  |  |  |  | | | |  | |  | | | | | | 0.11 | |
|  |  |  |  | | | |  | |  | | | | | |  | |
| Chemotherapy frequency*^1^ | q2w | 10 (47.6) | | | 21 (39.6) | | | | | | | | |  | | |
| (N=74） | q3w | 11 (52.4) | | | 32 (60.4) | | | | | | | | |  | | |
|  |  |  |  | | | |  | |  | | | | | | 0.52 | |
|  |  |  |  | | | |  | |  | | | | | |  | |
| Chemotherapy agent*^2^ | A/TC | 3 (14.3) | | | 10 (18.9) | | | | | | | | |  | | |
| (N=74) | AC-T/P | 8 (38.1) | | | 12 (22.6) | | | | | | | | |  | | |
|  | T/PA(C) | 6 (28.6) | | | 21 (39.6) | | | | | | | | |  | | |
|  | Platinum based | 2 (9.5) | | | 1 (1.9) | | | | | | | | |  | | |
|  | Standard^*3^ followed by Capecitabine | 2 (9.5) | | | 9 (17.0) | | | | | | | | | 0.31 | | |
|  |  |  |  | | | |  | |  | | | | | |  | |
|  |  |  |  | | | |  | |  | | | | | |  | |
| Radiotherapy | No | 7 (33.3) | | | 23 (42.6) | | | | | | | | |  | | |
|  | Yes | 14 (66.7) | | | 31 (57.4) | | | | | | | | |  | | |
|  |  |  |  | | | |  | |  | | | | | | 0.46 | |
|  |  |  |  | | | |  | |  | | | | | |  | |
| Radiotherapy dose (Gy) | 50–60 | 7 (50.0) | | | | | 10 (32.3) | | | | | | | |  | |
| (N=45) | 61–100 | 6 (42.9) | | | | | 8 (25.8) | | | | | | | |  | |
|  | >100 | 1 (7.1) | | | | | 13 (41.9) | | | | | | | |  | |
|  |  |  |  | | | |  | |  | | | | | | 0.06 | |
|  |  |  |  | | | |  | |  | | | | | |  | |
| Radiotherapy cycle | 25 | 10 (71.4) | | | | | 23 (74.2) | | | | | | | |  | |
| (N=45) | 30 | 4 (28.6) | | | | | 8 (25.8) | | | | | | | |  | |
|  |  |  |  | | | |  | |  | | | | | | 0.84 | |

IDC, invasive ductal carcinoma; LN, lymph node; LVI, lymphovascular invasion; ER, estrogen receptor; PR, progesterone receptor; EGFR, epidermal growth factor receptor; SLN, sentinel lymph node; ALND, axillary lymph node dissection; *1, q2w, every 2 weeks; q3w, every 3 weeks; *2, A, anthracyclines; C, cyclophosphamide; T, docetaxel; P, paclitaxel; *3 including AC-P, TAC, PA, and TA.

*P* values were derived from the Pearson’s Chi-square test, Fisher's exact test and Continuity Correction chi-square test.
