# Supplementary material for: Genomic Profiling Comparison of Germline BRCA and Non-BRCA Carriers Reveals CCNE1 Amplification as a Risk Factor for Non-BRCA Carriers in Patients With Triple-Negative Breast Cancer
Source: Front Oncol. 2020 Oct 30;10:583314. doi: 10.3389/fonc.2020.583314 (PMC7662137; doi:10.3389/fonc.2020.583314)
Supplement: Supplementary Table 2 — Events in BRCA1/2 mutation carriers and non-BRCA carriers. [file Table_2.DOCX]

**Table S2.** Events in *BRCA1/2* mutation carriers and non-*BRCA* carriers

| **Event** | *BRCA1/2* carriers (n=21) | | Non-*BRCA* carriers (n=54) | | | *P* |
| --- | --- | --- | --- | --- | --- | --- |
|  | N | % | | N | % |  |
| **DFS** | 5 | 23.8 | | 23 | 42.6 | 0.13 |
| **OS** | 4 | 19.0 | | 14 | 25.9 | 0.76 |

DFS, disease-free survival; OS, overall survival.
