# Supplementary material for: Genomic Profiling Comparison of Germline BRCA and Non-BRCA Carriers Reveals CCNE1 Amplification as a Risk Factor for Non-BRCA Carriers in Patients With Triple-Negative Breast Cancer
Source: Front Oncol. 2020 Oct 30;10:583314. doi: 10.3389/fonc.2020.583314 (PMC7662137; doi:10.3389/fonc.2020.583314)
Supplement: Supplementary Table 3, related to Table 3 — Comparison of somatic mutations between BRCA germline mutation carriers and non-carriers of triple-negative breast cancer (mutation frequency equal to or more than 4% in the whole cohort, p ≥ 0.05). [file Table_3.DOCX]

**Table S3, related to Table 3. Comparison of somatic mutations between *BRCA* germline mutation carriers and non-carriers of triple-negative breast cancer (mutation frequency equal to or more than 4% in the whole cohort, *p* value ≥ 0.05)**

|  | **mut (N=21)** | **wild (N=54)** | **Total (N=75)** | ***p*-value** |
| --- | --- | --- | --- | --- |
| *TP53* |  |  |  | 0.27 |
| notmut | 21 (100.0%) | 51 (94.4%) | 72 (96.0%) |  |
| wild | 0 (0.0%) | 3 (5.6%) | 3 (4.0%) |  |
| *MYC* |  |  |  | 0.43 |
| mut | 2 (9.5%) | 9 (16.7%) | 11 (14.7%) |  |
| wild | 19 (90.5%) | 45 (83.3%) | 64 (85.3%) |  |
| *PTEN* |  |  |  | 0.22 |
| mut | 1 (4.8%) | 8 (14.8%) | 9 (12.0%) |  |
| wild | 20 (95.2%) | 46 (85.2%) | 66 (88.0%) |  |
| wild | 20 (95.2%) | 52 (96.3%) | 72 (96.0%) |  |
| *MCL1* |  |  |  | 0.52 |
| mut | 3 (14.3%) | 5 (9.3%) | 8 (10.7%) |  |
| wild | 18 (85.7%) | 49 (90.7%) | 67 (89.3%) |  |
| *PTP4A3* |  |  |  | 0.39 |
| mut | 1 (4.8%) | 6 (11.1%) | 7 (9.3%) |  |
| wild | 20 (95.2%) | 48 (88.9%) | 68 (90.7%) |  |
| *GATA3* |  |  |  | 0.97 |
| mut | 2 (9.5%) | 5 (9.3%) | 7 (9.3%) |  |
| wild | 19 (90.5%) | 49 (90.7%) | 68 (90.7%) |  |
| *FAT3* |  |  |  | 0.76 |
| mut | 2 (9.5%) | 4 (7.4%) | 6 (8.0%) |  |
| wild | 19 (90.5%) | 50 (92.6%) | 69 (92.0%) |  |
| *RB1* |  |  |  | 0.21 |
| mut | 3 (14.3%) | 3 (5.6%) | 6 (8.0%) |  |
| wild | 18 (85.7%) | 51 (94.4%) | 69 (92.0%) |  |
| *EGFR* |  |  |  | 0.53 |
| mut | 2 (9.5%) | 3 (5.6%) | 5 (6.7%) |  |
| wild | 19 (90.5%) | 51 (94.4%) | 70 (93.3%) |  |
| *CCND2* |  |  |  | 0.68 |
| mut | 1 (4.8%) | 4 (7.4%) | 5 (6.7%) |  |
| wild | 20 (95.2%) | 50 (92.6%) | 70 (93.3%) |  |
| *NF1* |  |  |  | 0.83 |
| mut | 1 (4.8%) | 2 (3.7%) | 3 (4.0%) |  |
| wild | 20 (95.2%) | 52 (96.3%) | 72 (96.0%) |  |
| *KMT2C* |  |  |  | 0.89 |
| mut | 1 (4.8%) | 3 (5.6%) | 4 (5.3%) |  |
| wild | 20 (95.2%) | 51 (94.4%) | 71 (94.7%) |  |
| *CHD4* |  |  |  | 0.31 |
| mut | 2 (9.5%) | 2 (3.7%) | 4 (5.3%) |  |
| wild | 19 (90.5%) | 52 (96.3%) | 71 (94.7%) |  |
| *MTOR* |  |  |  | 0.89 |
| mut | 1 (4.8%) | 3 (5.6%) | 4 (5.3%) |  |
| wild | 20 (95.2%) | 51 (94.4%) | 71 (94.7%) |  |
| *PALB2* |  |  |  | 0.89 |
| mut | 1 (4.8%) | 3 (5.6%) | 4 (5.3%) |  |
| wild | 20 (95.2%) | 51 (94.4%) | 71 (94.7%) |  |
| *CCNE1* |  |  |  | 0.20 |
| mut | 0 (0.0%) | 4 (7.4%) | 4 (5.3%) |  |
| wild | 21 (100.0%) | 50 (92.6%) | 71 (94.7%) |  |
| *IKBKB* |  |  |  | 0.20 |
| mut | 0 (0.0%) | 4 (7.4%) | 4 (5.3%) |  |
| wild | 21 (100.0%) | 50 (92.6%) | 71 (94.7%) |  |
| *FGF6* |  |  |  | 0.89 |
| mut | 1 (4.8%) | 3 (5.6%) | 4 (5.3%) |  |
| wild | 20 (95.2%) | 51 (94.4%) | 71 (94.7%) |  |
| *FGF23* |  |  |  | 0.89 |
| mut | 1 (4.8%) | 3 (5.6%) | 4 (5.3%) |  |
| wild | 20 (95.2%) | 51 (94.4%) | 71 (94.7%) |  |
| *RUNX1* |  |  |  | 0.89 |
| mut | 1 (4.8%) | 3 (5.6%) | 4 (5.3%) |  |
| wild | 20 (95.2%) | 51 (94.4%) | 71 (94.7%) |  |
| *FGFR3* |  |  |  | 0.12 |
| mut | 2 (9.5%) | 1 (1.9%) | 3 (4.0%) |  |
| wild | 19 (90.5%) | 53 (98.1%) | 72 (96.0%) |  |
| *HSP90AA1* |  |  |  | 0.83 |
| mut | 1 (4.8%) | 2 (3.7%) | 3 (4.0%) |  |
| wild | 20 (95.2%) | 52 (96.3%) | 72 (96.0%) |  |
| *NOTCH1* |  |  |  | 0.12 |
| mut | 2 (9.5%) | 1 (1.9%) | 3 (4.0%) |  |
| wild | 19 (90.5%) | 53 (98.1%) | 72 (96.0%) |  |
| *NOTCH2* |  |  |  | 0.27 |
| mut | 0 (0.0%) | 3 (5.6%) | 3 (4.0%) |  |
| wild | 21 (100.0%) | 51 (94.4%) | 72 (96.0%) |  |
| *B4GALT3* |  |  |  | 0.27 |
| mut | 0 (0.0%) | 3 (5.6%) | 3 (4.0%) |  |
| wild | 21 (100.0%) | 51 (94.4%) | 72 (96.0%) |  |
| *BCOR* |  |  |  | 0.27 |
| mut | 0 (0.0%) | 3 (5.6%) | 3 (4.0%) |  |
| wild | 21 (100.0%) | 51 (94.4%) | 72 (96.0%) |  |
| *KIAA1549* |  |  |  | 0.83 |
| mut | 1 (4.8%) | 2 (3.7%) | 3 (4.0%) |  |
| wild | 20 (95.2%) | 52 (96.3%) | 72 (96.0%) |  |
| *DUSP6* |  |  |  | 0.83 |
| mut | 1 (4.8%) | 2 (3.7%) | 3 (4.0%) |  |
| wild | 20 (95.2%) | 52 (96.3%) | 72 (96.0%) |  |
| *MSH6* |  |  |  | 0.83 |
| mut | 1 (4.8%) | 2 (3.7%) | 3 (4.0%) |  |
| wild | 20 (95.2%) | 52 (96.3%) | 72 (96.0%) |  |
| *STAT4* |  |  |  | 0.83 |
| mut | 1 (4.8%) | 2 (3.7%) | 3 (4.0%) |  |
| wild | 20 (95.2%) | 52 (96.3%) | 72 (96.0%) |  |
| *WHSC1L1* |  |  |  | 0.27 |
| mut | 0 (0.0%) | 3 (5.6%) | 3 (4.0%) |  |
| wild | 21 (100.0%) | 51 (94.4%) | 72 (96.0%) |  |
| *ARID2* |  |  |  | 0.83 |
| mut | 1 (4.8%) | 2 (3.7%) | 3 (4.0%) |  |
| wild | 20 (95.2%) | 52 (96.3%) | 72 (96.0%) |  |
| *KMT2D* |  |  |  | 0.83 |
| mut | 1 (4.8%) | 2 (3.7%) | 3 (4.0%) |  |
| wild | 20 (95.2%) | 52 (96.3%) | 72 (96.0%) |  |
| *RAD52* |  |  |  | 0.83 |
| mut | 1 (4.8%) | 2 (3.7%) | 3 (4.0%) |  |
| wild | 20 (95.2%) | 52 (96.3%) | 72 (96.0%) |  |
| *NCOR1* |  |  |  | 0.27 |
| mut | 0 (0.0%) | 3 (5.6%) | 3 (4.0%) |  |
| wild | 21 (100.0%) | 51 (94.4%) | 72 (96.0%) |  |
| *EPHA5* |  |  |  | 0.27 |
| mut | 0 (0.0%) | 3 (5.6%) | 3 (4.0%) |  |
| wild | 21 (100.0%) | 51 (94.4%) | 72 (96.0%) |  |
| *RXRA* |  |  |  | 0.83 |
| mut | 1 (4.8%) | 2 (3.7%) | 3 (4.0%) |  |
| wild | 20 (95.2%) | 52 (96.3%) | 72 (96.0%) |  |
| *ARID1A* |  |  |  | 0.83 |
| mut | 1 (4.8%) | 2 (3.7%) | 3 (4.0%) |  |
| wild | 20 (95.2%) | 52 (96.3%) | 72 (96.0%) |  |

mut, mutation.

*P* values were derived from the Pearson’s Chi-square test, Fisher's exact test and Continuity Correction chi-square test.
