# Supplementary material for: Genomic Profiling Comparison of Germline BRCA and Non-BRCA Carriers Reveals CCNE1 Amplification as a Risk Factor for Non-BRCA Carriers in Patients With Triple-Negative Breast Cancer
Source: Front Oncol. 2020 Oct 30;10:583314. doi: 10.3389/fonc.2020.583314 (PMC7662137; doi:10.3389/fonc.2020.583314)
Supplement: Supplementary Table 4, related to Table 4 — Comparison of somatic mutant genes involved in the homologous recombination repair pathway between BRCA germline mutation carriers and non-carriers of triple-negative breast cancer (p ≥ 0.05). [file Table_4.DOCX]

**Table S4, related to Table 4. Comparison of somatic mutant genes involved in the homologous recombination repair pathway between *BRCA* germline mutation carriers and non-carriers of triple-negative breast cancer (p value ≥ 0.05)**

|  | **mut (N=21)** | **wild (N=54)** | **Total (N=75)** | ***p-*value** |
| --- | --- | --- | --- | --- |
| Total |  |  |  |  |
| mut | 3 (14.3%) | 12 (22.2%) | 15 (20.0%) | 0.53 |
| wild | 18 (85.7%) | 42 (77.8%) | 60 (80.0%) |  |
| *PALB2* |  |  |  | 0.89 |
| mut | 1 (4.8%) | 3 (5.6%) | 4 (5.3%) |  |
| wild | 20 (95.2%) | 51 (94.4%) | 71 (94.7%) |  |
| *RAD52* |  |  |  | 0.83 |
| mut | 1 (4.8%) | 2 (3.7%) | 3 (4.0%) |  |
| wild | 20 (95.2%) | 52 (96.3%) | 72 (96.0%) |  |
| *BRCA1* |  |  |  | 0.37 |
| mut | 0 (0.0%) | 2 (3.7%) | 2 (2.7%) |  |
| wild | 21 (100.0%) | 52 (96.3%) | 73 (97.3%) |  |
| *ATRX* |  |  |  | 0.37 |
| mut | 0 (0.0%) | 2 (3.7%) | 2 (2.7%) |  |
| wild | 21 (100.0%) | 52 (96.3%) | 73 (97.3%) |  |
| *ATM* |  |  |  | 0.53 |
| mut | 0 (0.0%) | 1 (1.9%) | 1 (1.3%) |  |
| wild | 21 (100.0%) | 53 (98.1%) | 74 (98.7%) |  |
| *BARD1* |  |  |  | 0.11 |
| mut | 1 (4.8%) | 0 (0.0%) | 1 (1.3%) |  |
| wild | 20 (95.2%) | 54 (100.0%) | 74 (98.7%) |  |
| *BRCA2* |  |  |  | 0.53 |
| mut | 0 (0.0%) | 1 (1.9%) | 1 (1.3%) |  |
| wild | 21 (100.0%) | 53 (98.1%) | 74 (98.7%) |  |
| *CHEK1* |  |  |  | 0.53 |
| mut | 0 (0.0%) | 1 (1.9%) | 1 (1.3%) |  |
| wild | 21 (100.0%) | 53 (98.1%) | 74 (98.7%) |  |
| *MRE11A* |  |  |  | 0.53 |
| mut | 0 (0.0%) | 1 (1.9%) | 1 (1.3%) |  |
| wild | 21 (100.0%) | 53 (98.1%) | 74 (98.7%) |  |
| *FANCL* |  |  |  | 0.53 |
| mut | 0 (0.0%) | 1 (1.9%) | 1 (1.3%) |  |
| wild | 21 (100.0%) | 53 (98.1%) | 74 (98.7%) |  |

mut, mutation

*P* values were derived from the Pearson’s Chi-square test, Fisher's exact test and Continuity Correction chi-square test.
