# Supplementary material for: Genomic Profiling Comparison of Germline BRCA and Non-BRCA Carriers Reveals CCNE1 Amplification as a Risk Factor for Non-BRCA Carriers in Patients With Triple-Negative Breast Cancer
Source: Front Oncol. 2020 Oct 30;10:583314. doi: 10.3389/fonc.2020.583314 (PMC7662137; doi:10.3389/fonc.2020.583314)
Supplement: Supplementary Table 5 — Univariate analysis of correlations between clinicopathological factors and genomic alterations and disease-free survival in non-BRCA carriers of triple-negative breast cancer. [file Table_5.DOCX]

**Table S5. Univariate analysis of correlations between clinicopathological factors and genomic alterations and disease-free survival in non-*BRCA* carriers of triple-negative breast cancer**

| **Factors** | **Hazard Ratio** | **95% CI** | ***p*-value** |
| --- | --- | --- | --- |
| Age | 0.73 | 0.49–1.08 | 0.11 |
| T stage | 2.18 | 1.24–3.83 | 0.007 |
| N stage | 1.39 | 0.98–1.97 | 0.06 |
| TNM stage | 2.52 | 1.14–5.55 | 0.02 |
| Lymph node status | 1.41 | 0.58–3.42 | 0.45 |
| Grade | 1.5 | 0.65–3.47 | 0.34 |
| LVI | 1.99 | 0.81–4.84 | 0.13 |
| Ki–67 | 0.35 | 0.05–2.62 | 0.30 |
| CK5/6 | 0.77 | 0.34–1.77 | 0.54 |
| EGFR (IHC) | 0.86 | 0.29–2.56 | 0.78 |
| Basal-like | 1.14 | 0.15–8.45 | 0.89 |
| TMB | 0.34 | 0.14–0.81 | 0.01 |
| CNV | 2.84 | 1.11–7.23 | 0.03 |
| Other gHRR mutation | 0.35 | 0.05–2.58 | 0.30 |
| *MYC* | 1.47 | 0.55–3.97 | 0.44 |
| *PTEN* | 0.73 | 0.22–2.46 | 0.61 |
| *MCL1* | 1.01 | 0.24–4.33 | 0.98 |
| *EPHA3* | 0.24 | 0.03–1.79 | 0.16 |
| *PTP4A3* | 1.92 | 0.65–5.66 | 0.24 |
| *GATA3* | 4.28 | 1.43–12.79 | 0.009 |
| *FAT3* | 0.5 | 0.07–3.74 | 0.50 |
| *RB1* | 1.86 | 0.43–8.01 | 0.40 |
| *PIK3CA* | 1.1 | 0.37–3.25 | 0.85 |
| *CCNE1* | 6.7 | 2.17–20.72 | 0.001 |
| *IKBKB* | 3.47 | 0.99–12.12 | 0.06 |
| *NOTCH2* | 1.69 | 0.4–7.22 | 0.47 |
| *B4GALT3* | 0.00 | 0–Inf | 0.99 |
| *BCOR* | 0.77 | 0.1–5.75 | 0.80 |
| *WHSC1L1* | 0.00 | 0–Inf | 0.99 |
| *NCOR1* | 2.16 | 0.5–9.28 | 0.29 |
| *EPHA5* | 2.42 | 0.56–10.34 | 0.23 |

LVI, lymphovascular invasion; EGFR, epidermal growth factor receptor; IHC, immunohistological chemistry; HRR, homologous recombination repair; TMB, tumor mutation burden; CNV, copy number variation; 95% CI, 95% confidence interval; gHRR mutation, mutated genes involved in the HRR pathway.
