# Supplementary material for: Genomic Profiling Comparison of Germline BRCA and Non-BRCA Carriers Reveals CCNE1 Amplification as a Risk Factor for Non-BRCA Carriers in Patients With Triple-Negative Breast Cancer
Source: Front Oncol. 2020 Oct 30;10:583314. doi: 10.3389/fonc.2020.583314 (PMC7662137; doi:10.3389/fonc.2020.583314)
Supplement: Supplementary Table 6 — Univariate analysis of correlations between clinicopathological factors and genomic alterations and overall survival in non-BRCA carriers of triple-negative breast cancer. [file Table_6.DOCX]

**Table S6. Univariate analysis of correlations between clinicopathological factors and genomic alterations and overall survival in non-*BRCA* carriers of triple-negative breast cancer**

| **Factors** | **Hazard Ratio** | **95% CI** | ***p*-value** |
| --- | --- | --- | --- |
| Age | 0.91 | 0.55–1.49 | 0.69 |
| T stage | 4.26 | 2.08–8.71 | <0.001 |
| N stage | 1.71 | 1.1–2.66 | 0.01 |
| TNM stage | 3.7 | 1.31–10.45 | 0.01 |
| Lymph node status | 1.53 | 0.48–4.89 | 0.47 |
| Grade | 1.47 | 0.5–4.29 | 0.48 |
| LVI | 2.1 | 0.7–6.26 | 0.18 |
| Ki–67 | 0.32 | 0.04–2.42 | 0.26 |
| CK5/6 | 0.75 | 0.26–2.16 | 0.59 |
| EGFR (IHC) | 0.43 | 0.13–1.44 | 0.17 |
| Basal-like | 0.64 | 0.08–4.88 | 0.66 |
| TMB | 0.42 | 0.14–1.25 | 0.12 |
| CNV | 3.47 | 0.97–12.45 | 0.06 |
| Other gHRR mutation | 0.57 | 0.07–4.37 | 0.58 |
| *MYC* | 2.14 | 0.67–6.83 | 0.19 |
| *PTEN* | 0.39 | 0.05–3.02 | 0.37 |
| *MCL1* | 2.02 | 0.45–9.05 | 0.36 |
| *EPHA3* | 0.44 | 0.06–3.34 | 0.42 |
| *PTP4A3* | 2.42 | 0.67–8.74 | 0.17 |
| *GATA3* | 1.87 | 0.42–8.39 | 0.41 |
| *FAT3* | 0.85 | 0.11–6.54 | 0.87 |
| *RB1* | 3.5 | 0.78–15.71 | 0.10 |
| *PIK3CA* | 1.47 | 0.41–5.26 | 0.55 |
| *CCNE1* | 2.58 | 0.58–11.58 | 0.21 |
| *IKBKB* | 2.87 | 0.64–12.95 | 0.17 |
| *NOTCH2* | 1.5 | 0.2–11.52 | 0.69 |
| *B4GALT3* | 0.00 | 0–Inf | 0.99 |
| *BCOR* | 1.13 | 0.15–8.62 | 0.90 |
| *WHSC1L1* | 0.00 | 0–Inf | 0.99 |
| *NCOR1* | 1.13 | 0.15–8.62 | 0.90 |
| *EPHA5* | 0.00 | 0–Inf | 0.99 |

LVI, lymphovascular invasion; EGFR, epidermal growth factor receptor; IHC, immunohistological chemistry; HRR, homologous recombination repair; TMB, tumor mutation burden; CNV, copy number variation; 95% CI, 95% confidence interval; gHRR mutation, mutated genes involved in the HRR pathway.
